# Future landscapes of women’s hormone-sensitive cancers: unraveling global trends, age stratification, and regional inequities (1990–2035)

**DOI:** 10.3389/fendo.2026.1691889

**Published:** 2026-05-01

**Authors:** Yi Zhou, Min Jiang, Yutao Wu, Jiao Wang, Xiaoyu Li, Ke Shen, Tao He, Chi Zhang, Hui Zong, Rongrong Wu, Rajeev K. Singla, Qing Lv, Bairong Shen

**Affiliations:** 1 Department of Breast Surgery and Institutes for Systems Genetics, Frontiers Science Center for Disease-Related Molecular Network, West China Hospital, Sichuan University, Chengdu, Sichuan, China; 2 Sichuan Academy of Chinese Medicine Sciences, Chengdu, Sichuan, China; 3 Department of Critical Care Medicine and Institutes for Systems Genetics, Frontiers Science Center for Disease-related Molecular Network, West China Hospital, Sichuan University, Chengdu, China; 4 Department of Computer Science and Information Technology, University of A Coruña, A Coruña, Spain; 5 School of Pharmaceutical Sciences, Lovely Professional University, Phagwara, Punjab, India

**Keywords:** breast cancer, cancer epidemiology, hormone-sensitive cancers, ovarian cancer, SDI inequality, uterine cancer

## Abstract

**Background:**

Hormone-sensitive cancers (HSCs) pose a significant threat to women’s health, with global rising incidence rates. This study provides the most up-to-date assessment of the global burden of HSCs.

**Methods:**

We retrieved HSC burden data from the Global Burden of Disease (GBD) study (1990–2021), categorized by age groups, and extracted risk factors linked to HSC mortality. Age-standardized incidence (ASIR), Age-standardized death (ASDR), and Age-standardized DALY rates (ASDiR), along with estimated annual percentage changes (EAPC), were used to evaluate HSC burden. Decomposition analysis identified contributing factors, while inequality and frontier analyses highlighted regional disparities and burden reduction potential across social development levels. The Bayesian Age-Period-Cohort (BAPC) model forecasted HSC burden trends to 2035.

**Results:**

From 1990 to 2021, the reproductive age group showed the fastest increase in ASIR for HSCs (EAPC = 0.44, 95% CI: 0.38–0.49), whereas the elderly adult group experienced a slight decline (EAPC = -0.07, 95% CI: -0.14–0.01). Both ASDR and ASDiR decreased across all age groups. For ASDR, the EAPCs were -0.52 (CI: -0.61–0.42) in reproductive age group, -0.61 (CI: -0.65–0.56) in pre-elderly adults, and -0.61 (CI: -0.65–0.56) in elderly adults. The corresponding EAPCs for ASDiR were -0.43 (CI: -0.52–0.33), -0.54 (CI: -0.59–0.50), and -0.61 (CI: -0.65–0.56), respectively. In 2021, breast cancer (BC) was the dominant HSC subtype, with ASIRs of 28.81 (UI: 26.84–30.94), 141.92 (UI: 132.94–152.15), and 194.89 (UI: 159.93–212.76) per 100,000 in reproductive, pre-elderly and elderly groups, respectively. ASIR increased with higher socio-demographic index (SDI), whereas ASDR and ASDiR rose initially then declined with increasing SDI. Dietary risks, alcohol use, and tobacco were the leading contributors to BC burden. The contribution of high fasting plasma glucose to BC increased globally among the reproductive-age women, while high body mass index (BMI) showed rising contribution to ovarian and uterine cancer burden in middle- to low-SDI regions. BAPC projections suggest that these trends are likely to continue through 2035.

**Conclusions:**

The global burden of HSCs continues to increase, with notable age and regional disparities in incidence and mortality. Lower SDI countries face faster-growing rates compared to higher SDI countries, a trend projected to persist through 2035.

## 1 Background

Hormone-sensitive cancers (HSCs) encompass a group of malignancies whose development and global burden are substantially influenced by endogenous and exogenous sex hormones. In this study, we focus on breast cancer (BC), ovarian cancer (OC), and uterine cancer (UC), aligning with established epidemiological classifications that group these as the primary female-specific, hormone-relative cancers (1, 2). The life-course risk of these cancers is intricately linked to hormonal milestones (e.g., menarche, pregnancy, and menopause), driving distinct age-related incidence trends (2, 3). Studies have shown that the incidence of BC rises markedly after menopause, with women aged over 50 experiencing a substantially higher prevalence compared with younger women (4, 5). In addition to mortality and morbidity, the treatment of HSCs may compromise reproductive capacity, particularly among younger patients receiving chemotherapy, endocrine therapy, or surgical interventions, which can have varying impacts on individuals, families, and even societal demographic development (6). Meanwhile, global demographic transitions, especially population ageing, are reshaping the epidemiology of chronic diseases, including cancer. The growing proportion of elderly individuals worldwide is expected to increase the absolute burden of HSCs and alter their age-specific patterns of incidence and mortality (7). Therefore, conducting stratified research on HSCs across different age groups and identifying disease progression patterns are of great significance for developing better prevention, control, and intervention strategies.

Compared to developed countries, women in developing countries face significant deficiencies in the screening, diagnosis, and treatment of HSCs (8). This has led to notable regional disparities in the incidence, mortality, and disability-adjusted life years (DALYs) associated with these cancers. For instance, the age-standardized rate (ASR) of BC for women under 50 in the Middle East and North Africa is 5.5 times lower than that of women over 50, and also lower than that of Western countries (9). Such disparities reflect not only differences in population age structures but also variations in health systems, screening programs, and health awareness. Beyond biological and healthcare factors, gender inequality plays a critical role in shaping the distribution and outcomes of women’s cancers worldwide. In many settings, sociocultural norms and economic dependence limit women’s autonomy in seeking medical care, which may delay early detection and contribute to poorer cancer outcomes (10, 11). Importantly, gender inequality is closely intertwined with broader socioeconomic development indicators such as the Socio-demographic Index (SDI). Countries with lower SDI levels often exhibit higher levels of gender inequality, weaker health systems, and reduced access to cancer screening and treatment services, thereby exacerbating disparities in cancer burden and survival outcomes (12). Recent research has highlighted the strong association between gender inequality and cancer burden among women, demonstrating that structural gender disparities contribute significantly to differences in incidence, mortality, and health outcomes across countries with varying SDI levels (13). Addressing gender inequality is therefore increasingly recognized as an essential component of global cancer control strategies.

The development of HSCs is associated with numerous risk factors, including genetic predisposition and environmental exposures. Genetic background encompasses factors such as family history of cancer, BRCA gene mutations, breast density, and body mass index (BMI) (14). Environmental exposures include smoking, alcohol consumption, diet, lifestyle, and exercise. These risk factors exhibit significant regional variations (6). For example, BMI is generally higher in developed countries compared to developing countries, contributing to different epidemiological trends of BC, UC, and OC across regions (15–17) These differences highlight the need for region-specific prevention strategies that address the unique risk profiles of populations.

Therefore, understanding the age-specific patterns, regional disparities, and structural determinants, including gender inequality and socioeconomic development, of HSCs are pivotal for advancing research and improving outcomes for women’s health. Comprehensive analyses that integrate demographic, social, and epidemiological perspectives can provide valuable evidence to guide targeted interventions, optimize healthcare resource allocation, and ultimately reduce the global burden of HSCs.

## 2 Methods

### 2.1 Overview

The data for this study includes incidence, deaths, and DALYs data for hormone-sensitive cancers (BC, OC, and UC, with ICD-10 codes listed in **Supplementary Table 1**) among women 15 years and older across 204 countries/territories, 21 GBD regions, and 5 SDI quintiles from 1990 to 2021, along with their respective 95% uncertainty interval (UI). Based on the WHO classification and epidemiological characteristics, the population was categorized into three age groups, 15–49 years (Reproductive ages, RA), 50–74 years (Pre-elderly adults, PA), and 75+ years (Elderly adults, EA)(https://www.who.int/data/gho/indicator-metadata-registry) (18). These data were accessed through the Global Burden of Disease Collaborative Network website (http://ghdx.healthdata.org), which provides publicly available results from the GBD database.

### 2.2 Risk factors

We also obtained level 2 risk factors associated with HSCs in GBD from 1990 to 2021. Specifically, risk factors for BC include alcohol use, dietary risks, high body-mass index, high fasting plasma glucose, low physical activity, and tobacco. Risk factors for OC include high body mass index and occupational risks. For UC, the risk factor is high body mass index (19).

### 2.3 Age-standardized rates

ASRs at the global, regional, and national levels were calculated using the formula:


$$ASR=\frac{\sum_{i=1}^{N}\alpha_{i}W_{i}}{\sum_{i=1}^{N}W_{i}}$$

Where *N* represents the total number of age groups, *i* denotes the *i*-th age group within *N*, $\alpha_{i}$ represents the rate of the *i*-th age group, and $W_{i}$ represents the number of individuals in the *i*-th age group. The 95% CI was determined by the *epitools* package in R software (20).

### 2.4 Estimated average percentage changes

EAPCs were used to calculate the rate of change in ASRs (ASIR, ASDR, and ASDiR) over a specific period from 1990 to 2021 (n=32 timepoints). Assuming a linear relationship between the natural logarithm of ASRs and the progression of years, this relationship can be summarized by the following equation:


y=α+βx+ϵ

Where *y* represents ln(ASR), *x* represents each calendar year, and *ϵ* represents the error term. Therefore, $\text{EAPC}=100\times (e^{\beta }-1)$, and the 95% CI was also derived based on the results of this linear regression. Statistical analyses were performed using R software (version 4.4.2).

### 2.5 Health inequality analysis

Health inequality analysis was conducted using the slope index and the concentration index of inequality. By regressing the incidence, mortality, and DALYs of HSCs across all age groups in different countries/territories against their relative positions on the SDI scale, the slope index of inequality was calculated. Based on the cumulative relative distribution of the population ranked by SDI and ASIR, ASDR, and ASDiR of HSCs, the Lorenz concentration curve was fitted, and the area under the curve was numerically integrated to calculate the health inequality concentration index (21).

### 2.6 Decomposition and frontier analysis

To quantify the contributions of different factors to the changes in ASIR and ASDR from 1990 to 2021, we employed a decomposition approach that partitions the total difference into components attributable to ageing, population growth, and epidemiological changes (22). In addition, the frontier analysis was applied to assess the relationship between the burden of HSCs and SDI. A non-linear frontier line was generated using free disposal hull analysis with 1000 bootstrap iterations (23, 24). The distance between the observed ASIR, ASDR, or ASDiR in a country/territory and its frontier line was defined as the effective difference, which was used to quantify the unrealized health gain under the current level of development in a country/territory.

### 2.7 Burden forecast through 2035

The BAPC model was applied to forecast the global and regional burden of HSCs from 1990 to 2035. Using the BAPC package in R, we predicted the ASIR, ASDR, and ASDiR for different regions up to 2035 (25). All statistical analyses and graphics were performed on R with version 4.4.2.

## 3 Results

### 3.1 Global burden of HSCs, 2021

#### 3.1.1 Global burden and trend of HSC in RA group (15–49 years)

**Figure 1:**
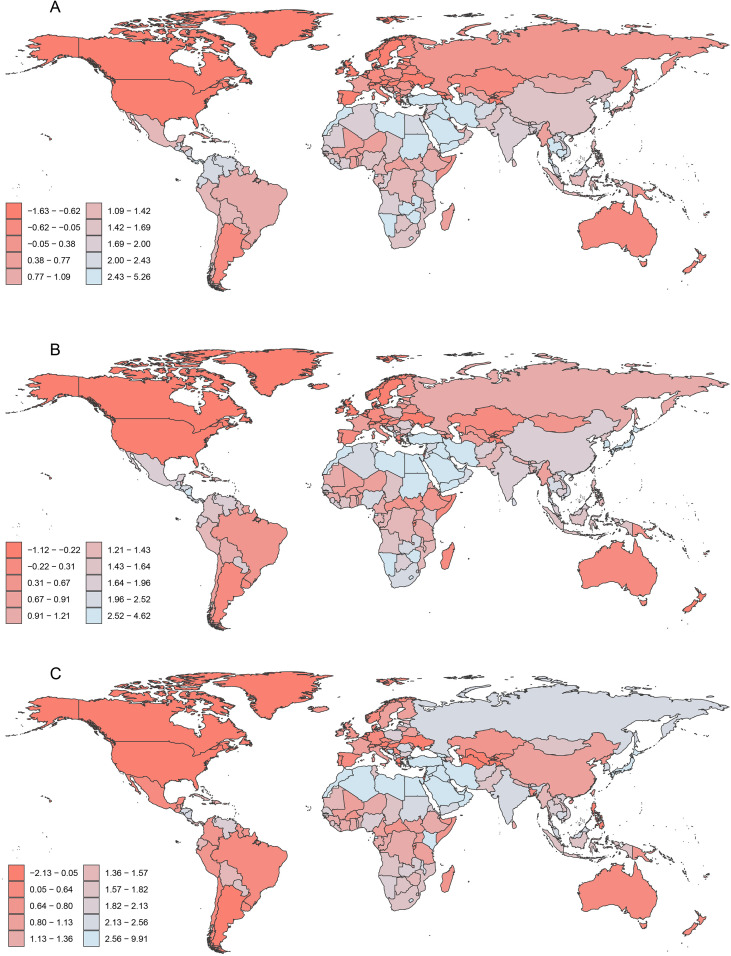
Global distribution of EAPC of ASIR for HSC among women of reproductive age, pre-elderly adults, and elderly adults in 2021. **(A)** The EAPC of ASIR for HSC in the reproductive age group. **(B)** The EAPC of ASIR for HSC in pre-elderly adults group. **(C)** The EAPC of ASIR for HSC in elderly adults group. EAPC, estimated annual percentage change; ASIR, age-standardized incidence rate; HSC, hormone-sensitive cancers.

Globally, the ASIR of HSCs per 100,000 populations increased from 29.08 (UI 27, 31.23) in 1990 to 34.65 (UI 31.59, 37.76) (**Table 1**) in 2021 in the reproductive-age group. In 2021, BC remained the predominant contributor to HSC incidence among women, followed by OC and UC, with ASIRs of 28.81 (UI 26.84, 30.94), 4.4 (UI 3.86, 4.88), and 3.02 (UI 2.6, 3.36), respectively (**Table 1**). Notably, there was a more than 13 times difference in HSC incidence between the country with the lowest rate (Niger 8.12; UI 4.36,13.76) and the highest rate (Monaco 117.25 [UI 72.32, 179.50]) in 2021 (**Supplementary Table 2**). From 1990 to 2021, the EAPC of ASIR for HSC was 0.44 (CI 0.38, 0.49) (**Table 1**). Of the three HSC subtypes, BC had the highest EAPC of 2.43 (CI 2.30, 2.56) (**Supplementary Figure 1A**), followed by UC and OC, with EAPCs of 2.42 (CI 2.19, 2.65) and 1.77 (CI 1.65, 1.89), respectively (**Supplementary Figures 2A**, **3A**). At national level, the EAPC of ASIR for HSC ranged from -1.60 (Armenia) to 5.21 (Turkey). Significant decreasing trends were primarily observed in countries such as Armenia, Ukraine, and Saint Kitts and Nevis, while the most rapid increases were concentrated in Turkey, Lesotho, and Saudi Arabia. Detailed EAPC values and their corresponding 95% CIs for all 204 countries and territories are provided in **Supplementary Table 2**, with the highest and lowest ranking countries highlighted in **Figure 1A**.

**Table 1:** The global and 5 SDI region burden of HSCs in reproductive age (15–49 years) group women.

Measure/region	Number of cases (UI), 1990	ASR (UI),1990	Number of cases (UI), 2021	ASR (UI), 2021	EAPC (CI), 1990-2021
Incidence					
HSCs	332578.78 (309676.44,356933.65)	29.08 (27,31.23)	706047.05 (763520.04,649080.85)	34.65 (31.59,37.76)	0.44 (0.38,0.49)
Breast cancer	256715.75 (246082.34,270223.8)	19.2 (18.4,20.21)	561438.09 (602977.82,523146.53)	28.81 (26.84,30.94)	2.43 (2.30,2.56)
Ovarian cancer	47982.63 (40523.31,55869.59)	3.59 (3.03,4.18)	85748.81 (95089.83,75168.93)	4.4 (3.86,4.88)	1.77 (1.65,1.89)
Uterine cancer	27880.4 (23070.78,30840.26)	2.08 (1.73,2.31)	58860.14 (65452.39,50765.39)	3.02 (2.6,3.36)	2.42 (2.19,2.65)
Low SDI	10419.39 (8263.81,12987.67)	12.04 (9.48,15.21)	42178.37 (49631.17,34948.03)	19.13 (15.57,22.87)	1.38 (1.24,1.52)
Low-middle SDI	30877.16 (26561.06,36481.33)	13.93 (11.78,16.57)	121336.29 (135577.02,107285.47)	25.71 (22.04,29.55)	1.92 (1.86,1.98)
Middle SDI	72818 (62798.11,83238.59)	20.24 (17.34,23.16)	230464.2 (255967.71,204257.61)	33.99 (29.81,38.18)	1.57 (1.50,1.64)
High-middle SDI	85844.23 (78579.47,93156.07)	34.41 (31.17,37.68)	158330.49 (180834.25,139000.31)	41.51 (35.89,48.23)	0.58 (0.50,0.66)
High SDI	132197.81 (129117.93,135220.47)	55.62 (53.56,57.7)	153071.78 (158483.88,147549.56)	51.98 (49.39,54.58)	-0.26 (-0.38,-0.14)
Deaths					
HSCs	99443.28 (91101.8,108221.63)	8.75 (8.02,9.55)	161826.78 (174911.28,148559.15)	7.93 (7.21,8.66)	-0.52 (-0.61,-0.42)
Breast cancer	78285.12 (73639.16,83738.85)	5.85 (5.51,6.26)	129405.59 (139007.9,120298.06)	6.64 (6.17,7.13)	1.45 (1.36,1.54)
Ovarian cancer	15490.34 (13203.64,17928.42)	1.16 (0.99,1.34)	25257.83 (27861.21,22277.54)	1.3 (1.14,1.43)	1.44 (1.33,1.55)
Uterine cancer	5667.82 (4259,6554.36)	0.42 (0.32,0.49)	7163.36 (8042.17,5983.55)	0.37 (0.31,0.41)	0.58 (0.38,0.78)
Low SDI	5760.47 (4637.42,7122.56)	6.78 (5.41,8.52)	18470.73 (21711.85,15412.84)	8.56 (7.03,10.19)	0.62 (0.51,0.73)
Low-middle SDI	14734.82 (12783.11,17132.32)	6.77 (5.8,7.96)	43021.34 (48150.66,38096.71)	9.18 (7.91,10.57)	0.92 (0.86,0.98)
Middle SDI	27391.22 (23820.19,31164.02)	7.77 (6.71,8.88)	54187.6 (60025.09,48366.41)	7.93 (7.02,8.86)	-0.13 (-0.23,-0.04)
High-middle SDI	25092.16 (22764.25,27453.03)	10.14 (9.15,11.18)	26816.12 (30094.46,23807.55)	6.96 (6.14,7.9)	-1.47 (-1.57,-1.37)
High SDI	26334.34 (25716.59,26886.06)	11.1 (10.73,11.43)	19169.39 (19796.04,18479.13)	6.46 (6.18,6.74)	-1.84 (-1.91,-1.78)
DALYs					
HSCs	5084302.1 (4636979.88,5550915.29)	441.64 (402.79,483.04)	8328137.17 (9000287.83,7641049.78)	409.57 (370.83,448.67)	-0.43 (-0.52,-0.33)
Breast cancer	3992794.45 (3744653.89,4279173.95)	298.56 (280,319.97)	6659459.91 (7145548.78,6192225.91)	341.71 (317.74,366.65)	1.48 (1.39,1.57)
Ovarian cancer	799648.14 (674906.17,931352.29)	59.79 (50.47,69.64)	1294995.57 (1431298.31,1139826.54)	59.79 (50.47,69.64)	1.42 (1.32,1.53)
Uterine cancer	291859.51 (217419.83,340389.05)	21.82 (16.26,25.45)	373681.68 (423440.74,308997.32)	21.82 (16.26,25.45)	0.63 (0.44,0.83)
Low SDI	292454.46 (234528.89,362184.36)	336.98 (267.84,423.15)	952372.4 (1122871.04,792747.56)	431.88 (353.63,515.94)	0.68 (0.57,0.79)
Low-middle SDI	750194.73 (649024.94,875410.17)	338.36 (288.65,399.17)	2206927.52 (2480721.67,1941623.73)	467.15 (400.45,541.28)	0.98 (0.92,1.04)
Middle SDI	1404317.14 (1215369.73,1602184.17)	390.42 (335.77,446.97)	2763134.71 (3051571.92,2462358.01)	407.44 (360.01,455.38)	-0.04 (-0.13,0.05)
High-middle SDI	1278060.03 (1156095.81,1403600.94)	509.76 (458.32,563.61)	1381064.5 (1556975.82,1230798.43)	363.34 (319.19,413.68)	-1.32 (-1.41,-1.23)
High SDI	1352703.44 (1308831.65,1397575.96)	569.01 (546.65,591.68)	1016434.01 (1072077.28,966231.4)	346.48 (327.02,366.91)	-1.70 (-1.76,-1.64)

SDI, socio-demographic index; HSCs, hormone-sensitive cancers; UI, uncertainty interval; ASR, age-standardized rate; CI, confidence interval; EAPC, estimated annual percentage change; DALYs, age-standardized disability-adjusted life years.

**Figure 2:**
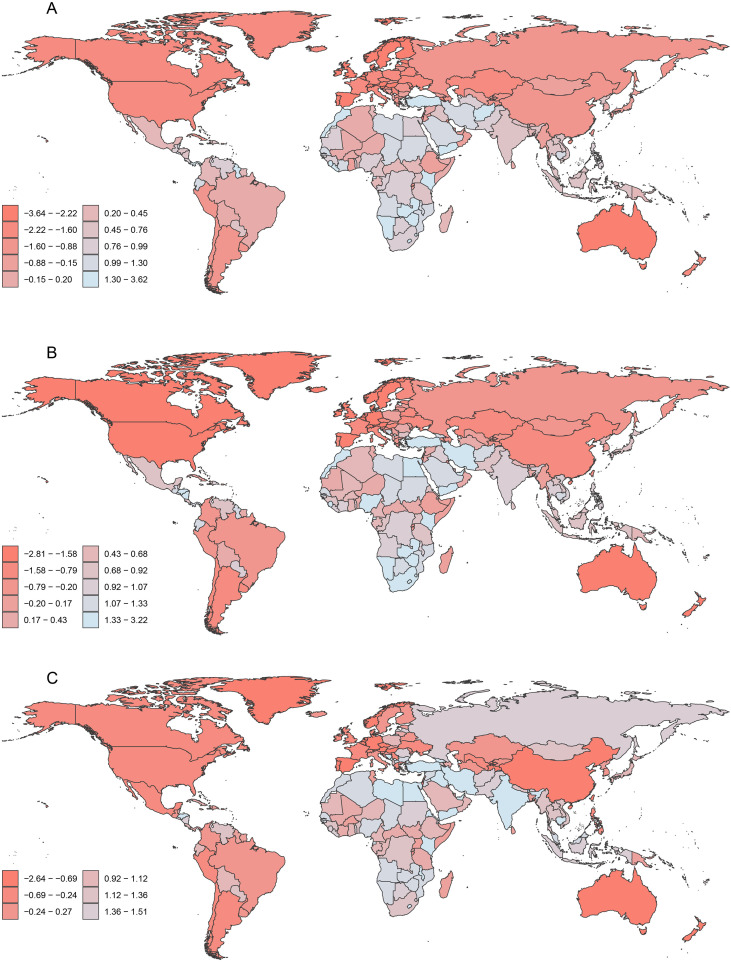
Global distribution of EAPC of ASDR for HSC among women of reproductive age, pre-elderly adults, and elderly adults in 2021. **(A)** The EAPC of ASDR for HSC in the reproductive age group. **(B)** The EAPC of ASDR for HSC in pre-elderly adults group. **(C)** The EAPC of ASDR for HSC in elderly adults group. EAPC, estimated annual percentage change; ASDR, age-standardized death rate; HSC, hormone-sensitive cancers.

The global ASDR of HSC decreased from 8.75 (UI 8.02, 9.55) in 1990 to 7.93 (UI 7.21, 8.66) in 2021, indicating an overall declining trend (**Table 1**). In 2021, BC remained the leading cause of HSC-related deaths, followed by OC and UC, with ASDRs of 6.64 (UI 6.17, 7.13), 1.30 (UI 1.14, 1.43), and 0.37 (UI 0.31, 0.41), respectively (**Table 1**). In addition, 69 countries had an ASDR above 10 per 100,000 population, while 13 countries/territories had an ASDR below 5. From 1990 to 2021, the EAPC of ASDR for HSC was -0.52 (CI -0.61, -0.42), showing an overall decrease (**Supplementary Table 2**; **Figure 2A**). The EAPCs for BC, UC, and OC were 1.45 (CI 1.36, 1.54), 1.44 (CI 1.33, 1.55), and 0.58 (CI 0.38, 0.78), respectively (**Table 1**; **Supplementary Figures 4A**-**6A**). Among these, 91 countries/territories showed a decreasing trend, while 114 countries/territories exhibited an increasing trend (**Supplementary Table 2**, **Figure 2A**).

**Figure 3:**
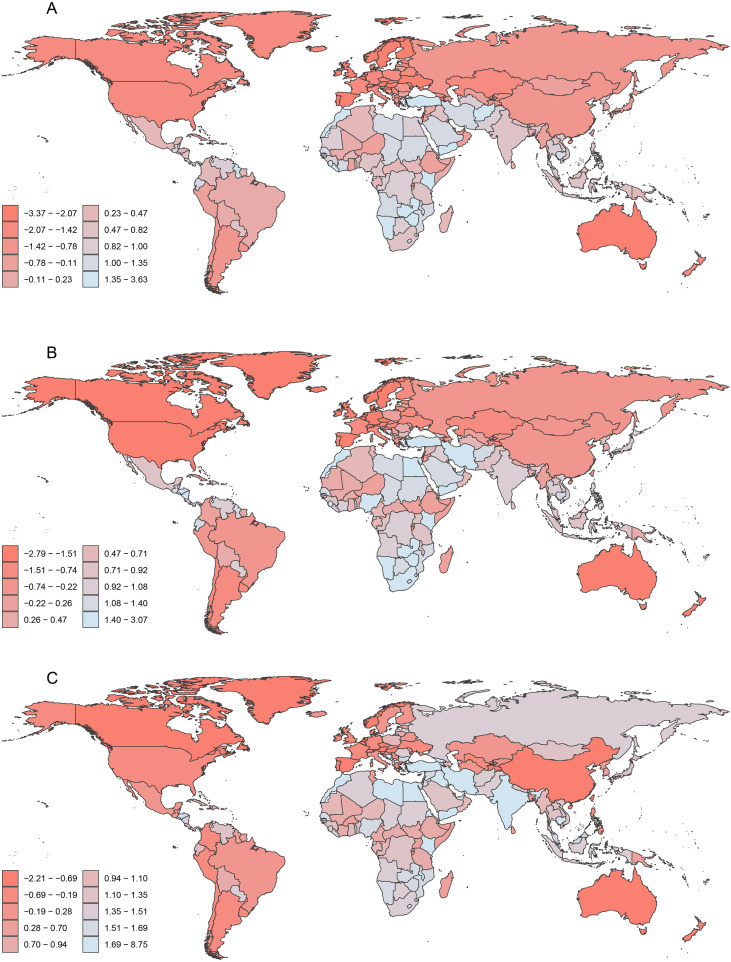
Global distribution of EAPC of ASDiR for HSC among women of reproductive age, pre-elderly adults, and elderly adults in 2021. **(A)** The EAPC of ASDiR for HSC in the reproductive age group. **(B)** The EAPC of ASDiR for HSC in pre-elderly adults group. **(C)** The EAPC of ASDiR for HSC in elderly adults group. EAPC, estimated annual percentage change; ASDiR, age-standardized disability-adjusted life years (DALYs) rate; HSC, hormone-sensitive cancers.

The global ASDiR of HSC declined from 441.64 (UI 402.79, 483.04) in 1990 to 409.57 (UI 370.83, 448.67) in 2021, showing an overall decreasing trend (**Table 1**). In 2021, BC was the leading contributor to HSC-related DALYs, followed by OC and UC, with ASDiRs of 341.71 (UI 317.74, 366.65), 59.79 (UI 50.47, 69.64), and 21.82 (UI 16.26, 25.45), respectively (**Table 1**). From 1990 to 2021, the EAPC in ASDiR for HSC was -0.43 (CI -0.52 to -0.33), indicating an overall declining trend. The EAPCs for BC, UC, and OC were 1.48 (CI 1.39, 1.57), 1.42 (CI 1.32, 1.53), and 0.63 (CI 0.44, 0.83), respectively (**Table 1**; **Supplementary Figures 7A**-**9A**). Among these, 87 countries showed a decreasing trend, while 118 countries exhibited an increasing trend (**Supplementary Table 2**; **Figure 3A**).

#### 3.1.2 Global burden of HSC in PA group (50–74 years)

In the PA group, the global ASIR of HSC increased from 176.22 (UI 166.29, 185.66) in 1990 to 201.31 (UI 185.12, 217.58) in 2021, significantly exceeding the rate in the PA group (**Table 2**). In 2021, BC remained the predominant contributor to HSC incidence among women in the PA group, followed by UC and OC, with ASIRs of 141.92 (UI 132.94, 152.15), 40.35 (UI 37.23, 43.71), and 19.72 (UI 18.04, 21.43), respectively (**Table 2**). The difference between the country with the lowest HSC incidence rate (Niger: 60.91; [UI 36.25, 95.52]) and the highest (United Arab Emirates: 614.89; [UI 387.81, 929.11]) in 2021 exceeded 9 times (**Supplementary Table 3**). From 1990 to 2021, the EAPC of ASIR for HSC was 0.40 (CI 0.36, 0.44) (**Table 2**). Among the three HSCs, BC had the highest EAPC at 3.04 (CI 2.99, 3.09) (**Supplementary Figure 1B**), followed by OC at 3.17 (CI 3.01, 3.34) and UC at 1.98 (CI 1.91, 2.05) (**Supplementary Figures 2B**, **3B**). Additionally, 180 countries exhibited an increasing trend in EAPC of ASIR for HSC, while only 25 countries showed a decline (**Supplementary Table 3**). The EAPC values ranged from a minimum of -1.11 (Greenland) to a maximum of 4.57 (Egypt). Significant downward trends were primarily concentrated in high-income regions, including Greenland, Denmark, and Bermuda, whereas the most rapid escalations were observed in the Middle East and North Africa, led by Egypt, Turkey, and Saudi Arabia. The detailed ranking and comprehensive EAPC data for all countries are provided in **Supplementary Table 3**, with the extreme-trend locations explicitly highlighted in the global heatmap (**Figure 1B**).

**Table 2:** The global and 5 SDI region burden of HSCs in pre-elderly adults age (50–74 years) group women.

Measure/region	Number of cases (UI), 1990	ASR (UI),1990	Number of cases (UI), 2021	ASR (UI), 2021	EAPC (CI), 1990-2021
Incidence					
HSCs	692901.85 (658392.71,724507.07)	176.22 (166.29,185.66)	1700638.5 (1829435.05,1584536.87)	201.31 (185.12,217.58)	0.40 (0.36,0.44)
Breast cancer	472851.51 (453313.52,490466.58)	120.44 (115.46,124.92)	1194865.97 (1280984.46,1119235.06)	141.92 (132.94,152.15)	3.04 (2.99,3.09)
Ovarian cancer	87458.14 (81714.83,94671.7)	22.28 (20.81,24.11)	166032.42 (180447.19,151876.88)	19.72 (18.04,21.43)	1.98 (1.91,2.05)
Uterine cancer	132592.2 (123364.36,139368.8)	33.77 (31.42,35.5)	339740.12 (368003.4,313424.93)	40.35 (37.23,43.71)	3.17 (3.01,3.34)
Low SDI	16021.83 (12900.96,19680.96)	71.86 (56.77,88.89)	52416.91 (60670.49,44603.56)	102.77 (85.84,120.68)	1.11 (0.97,1.25)
Low-middle SDI	39437.39 (33792.16,46667.06)	65.73 (55.34,78.88)	172664.19 (196096.01,152451.14)	118.42 (103.25,136.76)	1.92 (1.86,1.98)
Middle SDI	93734.88 (82831.97,105748.35)	89.49 (78.15,101.89)	419135.35 (472092.2,369971.19)	150.05 (131.1,170.96)	1.55 (1.49,1.60)
High-middle SDI	196457.72 (186616.41,206250.9)	188.44 (176.51,200.43)	456595.2 (509387.34,411293.44)	232.56 (206.97,263.32)	0.57 (0.50,0.64)
High SDI	346321.1 (333339.44,354491.7)	335.87 (319.8,349.06)	597934.73 (620955.73,559405.42)	343.21 (319.48,361.52)	0.12 (0.02,0.22)
Deaths					
HSCs	284197.19 (266891.65,302039.35)	72.33 (67.66,77.14)	528288.47 (570147.64,489124.31)	62.36 (57.17,67.62)	-0.61 (-0.65,-0.56)
Breast cancer	189790.79 (180233.04,199748.02)	48.34 (45.91,50.88)	358372.01 (383602.46,334339.85)	42.57 (39.71,45.56)	1.97 (1.87,2.08)
Ovarian cancer	60964.36 (56751.6,66202.41)	15.53 (14.45,16.86)	111336.41 (121219.91,101917.06)	13.22 (12.11,14.4)	1.81 (1.72,1.90)
Uterine cancer	33442.04 (29907.01,36088.92)	8.52 (7.62,9.19)	58580.05 (65325.27,52867.4)	6.96 (6.28,7.76)	1.63 (1.45,1.81)
Low SDI	11916.52 (9603.57,14619.11)	54.01 (42.83,66.83)	33722.67 (38930.24,28676.65)	67 (55.97,78.33)	0.66 (0.55,0.77)
Low-middle SDI	26428.42 (22640.76,31475.56)	44.55 (37.4,53.67)	91841.4 (104666.5,80971.71)	63.43 (55.21,73.46)	1.15 (1.09,1.20)
Middle SDI	52088.75 (46136.1,58971.01)	50.07 (43.8,57.24)	152127.76 (171121.24,135349.07)	54.8 (48.2,61.94)	0.11 (0.04,0.18)
High-middle SDI	83630.96 (78908.34,87889.33)	80.13 (74.97,85.2)	125876.31 (139347.64,114106.74)	63.31 (56.9,70.69)	-1.00 (-1.10,-0.89)
High SDI	109711.37 (106037.2,112405.41)	105.07 (100.67,108.63)	124073.57 (129048.36,116537.09)	69.09 (64.63,72.38)	-1.39 (-1.42,-1.36)
DALYs					
HSCs	8640257.12 (8097433.66,9194379.98)	2198.66 (2052.17,2355.01)	16244056.62 (17578320.49,15010822.61)	1926.87 (1766.07,2094.68)	-0.54 (-0.59,-0.50)
Breast cancer	5873649.16 (5570300.68,6189889.22)	1496.04 (1418.78,1576.59)	11259634.42 (12100179.43,10469846.23)	1337.39 (1243.58,1437.22)	2.08 (1.99,2.18)
Ovarian cancer	1779412.66 (1656470.15,1938007.63)	453.22 (421.91,493.62)	3228178.22 (3521762.8,2961996.5)	383.43 (351.82,418.3)	1.85 (1.77,1.93)
Uterine cancer	987195.31 (870662.82,1066483.13)	251.44 (221.76,271.64)	1756243.99 (1956378.25,1578979.88)	208.6 (187.55,232.37)	1.77 (1.61,1.94)
Low SDI	363043.01 (291140.23,448302.33)	1608.57 (1272.05,2000.61)	1028745.1 (1189989.01,872695.72)	1994.16 (1666.79,2334.01)	0.65 (0.53,0.76)
Low-middle SDI	816041.52 (697492.66,976034.9)	1346.28 (1130.96,1624.24)	2821735.44 (3225367.66,2485783.65)	1927.07 (1672.74,2233.62)	1.17 (1.11,1.22)
Middle SDI	1611404.73 (1426470.23,1829928.75)	1532.14 (1337.04,1755.34)	4754496.07 (5360945.36,4209287.07)	1698.71 (1492.28,1920.07)	0.15 (0.09,0.22)
High-middle SDI	2552505.58 (2403515.41,2690942.88)	2452.87 (2289.61,2618.3)	3841729.96 (4282517.09,3473116.79)	1960.6 (1756.46,2200.88)	-0.95 (-1.05,-0.85)
High SDI	3284560.33 (3165089.59,3399063.71)	3225.64 (3081.45,3361.26)	3778172.78 (3986695.51,3543293.71)	2184.49 (2036.6,2322.53)	-1.28 (-1.32,-1.25)

SDI, socio-demographic index; HSCs, hormone-sensitive cancers; UI, uncertainty interval; ASR, age-standardized rate; CI, confidence interval; EAPC, estimated annual percentage change; DALYs, age-standardized disability-adjusted life years.

The global ASDR of HSC decreased from 72.33 (UI 67.66, 77.14) in 1990 to 62.36 (UI 57.17, 67.62) in 2021, remaining significantly higher than that of the PA age group (**Table 2**). In 2021, BC continued to be the primary cause of HSC-related mortality among women in the PA group, followed by OC and UC, with ASDR values of 42.57 (UI 39.71, 45.56), 13.22 (UI 12.11, 14.40), and 6.96 (UI 6.28, 7.76), respectively (**Table 2**). 49 countries had an HSC mortality rate over 100 per 100,000 population, while 26 countries had a mortality rate below 50 per 100,000 population in 2021. From 1990 to 2021, the EAPC of ASDR for HSC was -0.61 (CI -0.65, -0.56), indicating a decreasing trend (**Supplementary Table 3**). The EAPCs for BC, UC, and OC were 1.97 (CI 1.87, 2.08) (**Supplementary Figure 4B**), 1.81 (CI 1.72, 1.90) (**Supplementary Figure 5B**), and 1.63 (CI 1.45, 1.81) (**Supplementary Figure 6B**), respectively (**Table 2**). A decreasing trend for EAPC was observed in 73 countries, while an increasing trend was noted in 132 countries (**Supplementary Table 3**, **Figure 2B**).

The global ASDiR of HSC decreased from 2198.66 (UI 2052.17, 2355.01) in 1990 to 1926.87 (UI 1766.07, 2094.68) in 2021 (**Table 2**). In 2021, BC remained the leading contributor to the ASDiR of HSC among women in the PA group, followed by OC and UC, with ASDiR values of 1337.39 (UI 1243.58, 1437.22), 383.43 (UI 351.82, 418.3), and 208.6 (UI 187.55, 232.37), respectively (**Table 2**). From 1990 to 2021, the EAPC of ASDiR for HSC was -0.54 (CI -0.59, -0.50), indicating a decreasing trend. The EAPCs for BC, UC, and OC were 2.08 (CI 1.99, 2.18) (**Supplementary Figure 7B**), 1.85 (CI 1.77, 1.93) (**Supplementary Figure 8B**), and 1.77 (CI 1.61, 1.94) (**Supplementary Figure 9B**), respectively (**Table 2**). A decreasing trend in the EAPC of ASDiR was observed in 73 countries, whereas 132 countries exhibited an increasing trend (**Supplementary Table 3**; **Figure 3B**).

#### 3.1.3 Global burden of HSC in EA group (75+ years)

In the EA group, the global ASIR of HSC was the highest among the three age groups, increasing from 266 (UI 232.64, 284.41) in 1990 to 267.47 (UI 220.47, 293.92) in 2021 (**Table 3**). In 2021, BC remained the leading cause of HSC incidence among women in the EA age group, followed by OC and UC, with ASIRs of 194.89 (UI 159.93, 212.76), 44.78 (UI 37.41, 49.01), and 28.12 (UI 23.51, 30.92), respectively (**Table 3**). The difference between the country with the lowest (Bangladesh: 31.45 [UI 17.47, 56.45]) and highest (United Arab Emirates: 1708.81 [UI 1105.35, 2523.94]) ASIR of HSC in 2021 exceeded 53 times (**Supplementary Table 4**). The EAPC of ASIR for HSC from 1990 to 2021 was -0.07 (CI -0.14, 0.01). Among HSCs, UC had the highest EAPC of 3.15 (CI 3.09, 3.22), followed by BC and OC at 2.94 (CI 2.89, 3.00) and 2.34 (CI 2.20, 2.47), respectively (**Table 3**; **Supplementary Figures 1C**-**3C**). Additionally, 186 countries experiencing upward trends in EAPC of ASIR for HSC, while only 19 countries showed a decline (**Supplementary Table 4**). The EAPC values spanned a broad range, from a minimum of -2.11(-2.89,-1.33) (Guam) to a maximum of 9.81(8.66,10.96) (United Arab Emirates). Significant decreasing trends were limited to a small number of locations, most notably Guam, Greenland, and San Marino. In contrast, the vast majority of nations exhibited sustained growth in the incidence burden, as detailed in the comprehensive country-level data in **Supplementary Table 4**. The countries with the most pronounced increasing and decreasing trends have been explicitly highlighted in the global heatmap (**Figure 1C**).

**Table 3:** The global and 5 SDI region burden of HSCs in elderly adults (75+ years) group women.

Measure/region	Number of cases (UI), 1990	ASR (UI),1990	Number of cases (UI), 2021	ASR (UI), 2021	EAPC (CI), 1990-2021
Incidence					
HSCs	190786.89 (168833.22,201918.07)	266 (232.64,284.41)	448541.32 (490227.35,369923.27)	267.47 (220.47,293.92)	-0.07 (-0.14,0.01)
Breast cancer	136313.45 (120335.62,144112.39)	188.68 (166.56,199.47)	326432.96 (356359.96,267878.3)	194.89 (159.93,212.76)	2.94 (2.89,3.00)
Ovarian cancer	23655.2 (21016.14,25161.07)	32.74 (29.09,34.83)	47094.77 (51784.97,39379.46)	28.12 (23.51,30.92)	2.34 (2.20,2.47)
Uterine cancer	30818.25 (27481.46,32644.62)	42.66 (38.04,45.19)	75013.59 (82082.43,62665.51)	44.78 (37.41,49.01)	3.15 (3.09,3.22)
Low SDI	2055.73 (1636.71,2439.41)	87.69 (68.25,106.41)	8141.14 (9324.26,6909.05)	135.65 (111.97,158.61)	1.44 (1.30,1.58)
Low-middle SDI	4937.97 (4128.94,5773.95)	69.49 (57.29,82.85)	24538.1 (28434.3,21438.33)	115.1 (98.23,134.87)	1.62 (1.54,1.70)
Middle SDI	13018.96 (11547.13,14336.7)	96.31 (83.95,107.63)	61422.99 (69186.23,52306.73)	136.04 (114.06,155.01)	0.99 (0.92,1.06)
High-middle SDI	40737.16 (36809.7,43095.92)	200.11 (177.71,214.27)	107568.44 (118244.5,89874.43)	257.88 (214.94,286.6)	0.86 (0.73,0.98)
High SDI	129801.08 (113551.97,138334.1)	451.37 (391.9,486.15)	246286.29 (271443.03,197322.93)	465.27 (376.7,516.21)	-0.03 (-0.09,0.03)
Deaths					
HSCs	122370.02 (108998.29,129644.5)	177.83 (155.68,190.71)	254090.81 (279077.68,211047.63)	150.41 (124.68,165.88)	-0.61 (-0.65,-0.56)
Breast cancer	82501.58 (73424.91,87103.43)	114.19 (101.63,120.56)	173147.7 (189710.06,142885.59)	103.37 (85.31,113.26)	2.51 (2.47,2.55)
Ovarian cancer	24129.41 (21522.96,25670.98)	33.4 (29.79,35.53)	49014.44 (53912.06,41225.52)	29.26 (24.61,32.19)	2.42 (2.29,2.54)
Uterine cancer	15739.03 (14050.42,16870.09)	21.79 (19.45,23.35)	31928.67 (35455.56,26936.52)	19.06 (16.08,21.17)	2.49 (2.42,2.57)
Low SDI	2194.8 (1759.63,2598.86)	97.33 (75.72,118.49)	8192.2 (9405.28,6964.95)	142.06 (117.32,166.34)	1.25 (1.12,1.39)
Low-middle SDI	5000.03 (4168.35,5863.47)	72.76 (59.65,87.32)	22082.56 (25797.71,19236.31)	106.52 (90.4,125.79)	1.23 (1.14,1.32)
Middle SDI	11933.03 (10568.1,13145.85)	92.53 (80.71,103.85)	43598.6 (49201.45,37033.78)	98.44 (82.64,112.19)	0.05 (-0.04,0.15)
High-middle SDI	30088.98 (27240.91,31809.22)	155.6 (138.05,166.8)	63541.01 (69690.13,53056.77)	150.58 (126.03,166.72)	-0.14 (-0.27,-0.01)
High SDI	72966.01 (63799.8,77815.64)	258.25 (223.54,278.84)	116276.4 (129019.85,93050.7)	205.36 (164.63,228.41)	-0.82 (-0.88,-0.77)
DALYs					
HSCs	1722510.9 (1552218.78,1831214.63)	2398.05 (2126.87,2570.94)	3408807.11 (3739840.33,2902666.66)	2031.53 (1717.29,2236.73)	-0.61 (-0.65,-0.56)
Breast cancer	1169958.34 (1054616.38,1241807.66)	1619.4 (1459.75,1718.85)	2335707.28 (2554407.94,1990174.84)	1394.48 (1188.18,1525.05)	2.37 (2.33,2.41)
Ovarian cancer	330174.77 (297222.68,350851.16)	457.01 (411.4,485.63)	640082.52 (704511.5,544239.35)	382.15 (324.92,420.61)	2.28 (2.14,2.41)
Uterine cancer	222377.8 (200379.72,238555.8)	307.81 (277.36,330.2)	433017.31 (480920.89,368252.47)	258.52 (219.86,287.12)	2.38 (2.30,2.46)
Low SDI	31339.76 (25114.54,37229.05)	1295.57 (1015.82,1567.26)	113589.28 (130590.06,96985.54)	1855.35 (1544.78,2167.57)	1.20 (1.07,1.32)
Low-middle SDI	70659.53 (59201.73,82987.5)	973.21 (803.34,1163.39)	305602.28 (357434.36,266580.1)	1420.22 (1213.98,1674.12)	1.19 (1.11,1.28)
Middle SDI	167464.32 (148532.67,185371.21)	1217.32 (1067.03,1365.83)	596480.13 (672496.27,513117.89)	1321.69 (1120.76,1502.24)	0.12 (0.03,0.20)
High-middle SDI	429712.88 (390688.48,455400.11)	2106.92 (1890.21,2254.43)	849184.1 (932678.02,729842.8)	2037.58 (1737.62,2255.13)	-0.14 (-0.26,-0.01)
High SDI	1020736.99 (906094.49,1091149.45)	3559.97 (3125.63,3845.03)	1538693.42 (1705589.72,1254933.48)	2863.63 (2349.12,3187.74)	-0.80 (-0.86,-0.75)

SDI, socio-demographic index; HSCs, hormone-sensitive cancers; UI, uncertainty interval; ASR, age-standardized rate; CI, confidence interval; EAPC, estimated annual percentage change; DALYs, age-standardized disability-adjusted life years.

The global ASDR for HSC decreased from 177.83 (UI 155.68, 190.71) in 1990 to 150.41 (UI 124.68, 165.88) in 2021, remaining the highest among the three age groups (**Table 3**). In 2021, BC remained the leading cause of HSC-related deaths in elderly women, followed by OC and UC, with ASDRs of 103.37 (UI 85.31, 113.26), 29.26 (UI 24.61, 32.19), and 19.06 (UI 16.08, 21.17), respectively(**Table 3**). 150 countries had HSC mortality rates reaching 100 per 100,000 population, while only 2 countries had rates below 50 (**Supplementary Table 4**; **Figure 2C**). The EAPC of ASDR for HSC from 1990 to 2021 was -0.61 (CI -0.65, -0.56), indicating a decline. For BC, UC, and OC, the EAPCs of ASDR were 2.51 (CI 2.47, 2.55), 2.42 (CI 2.29, 2.54), and 2.49 (CI 2.42, 2.57), respectively (**Table 3**; **Supplementary Figures 4C**-**6C**). Among countries, 151 exhibited increasing in EAPC of HSC ASDR, while only 54 showed declining trends (**Supplementary Table 4**; **Figure 2C**).

The global ASDiR of HSC declined from 2398.05 (UI 2126.87, 2570.94) in 1990 to 2031.53 (UI 1717.29, 2236.73) in 2021 (**Table 3**). In 2021, BC remained the primary contributor to HSC ASDiR in elderly women, followed by OC and UC, with ASDiRs of 1394.48 (UI 1188.18, 1525.05), 382.15 (UI 324.92, 420.61), and 258.52 (UI 219.86, 287.12), respectively (**Table 3**). The EAPC of ASDiR for HSC from 1990 to 2021 was -0.61 (CI -0.65, -0.56), reflecting a decreasing trend. For OC, BC, and UC, the EAPCs were 2.38 (CI 2.30, 2.46), 2.37 (CI 2.33, 2.41), and 2.28 (CI 2.14, 2.41), respectively (**Table 3**; **Supplementary Figures 7C**-**9C**). Among countries, 151 showed increasing in EAPC of ASDiR for HSC, while 54 exhibited declining trends (**Supplementary Table 4**; **Figure 3C**).

### 3.2 Regional burden and trend of HSCs, 2021

#### 3.2.1 Regional burden and trend of HSC in RA group (15–49 years)

**Figure 4:**
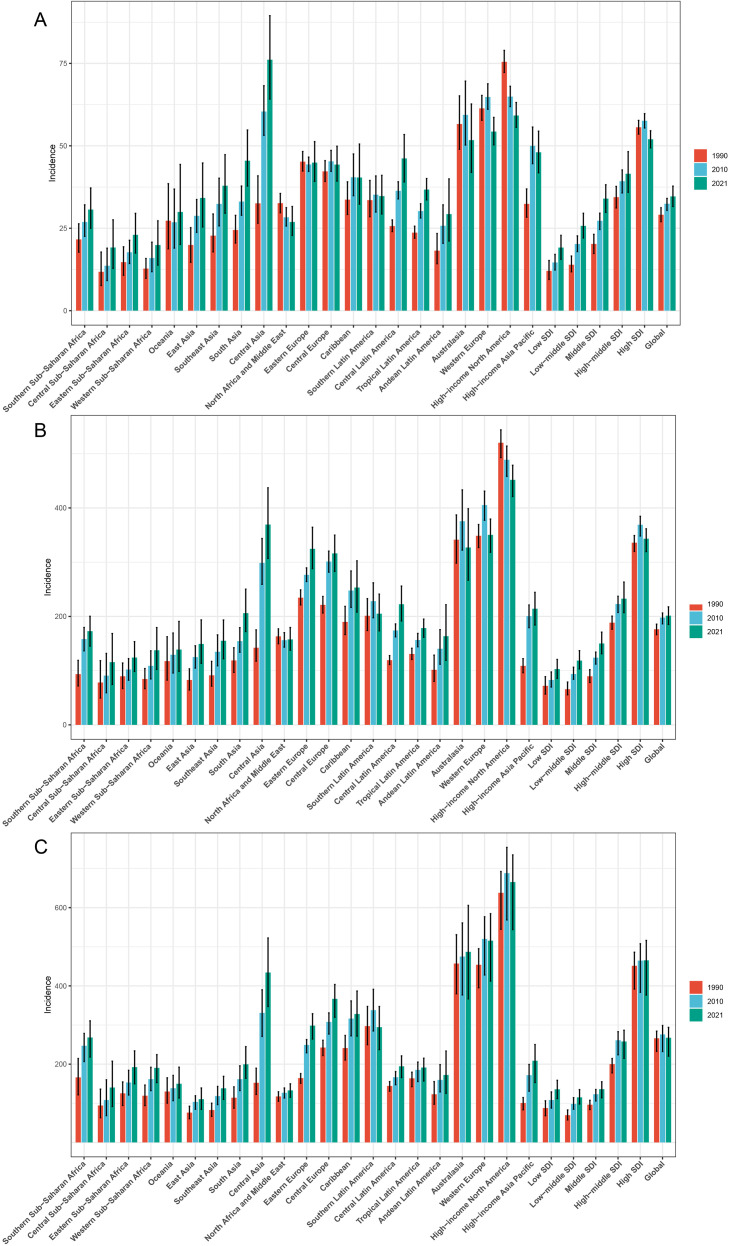
Regional ASIR barplot for HSC among women of reproductive age, pre-elderly adults, and elderly adults in 1990, 2010, and 2021. **(A)** The ASIR for HSC in the reproductive age group. **(B)** The ASIR for HSC in pre-elderly adults group. **(C)** The ASIR for HSC in elderly adults group. ASIR, age-standardized incidence rate; HSC, hormone-sensitive cancers.

**Figure 5:**
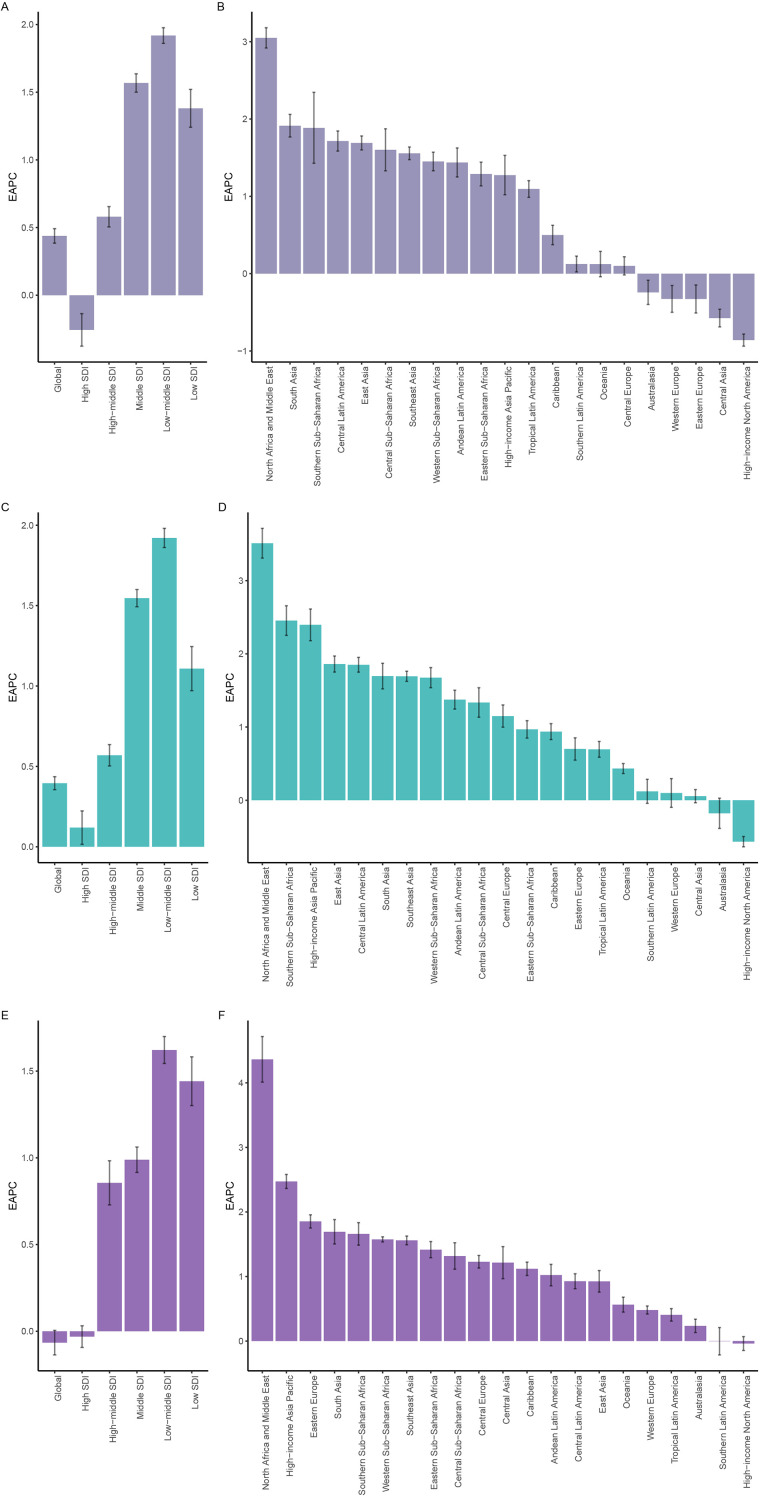
The EAPC of ASIR for HSC among women of reproductive age, pre-elderly adults, and elderly adults across 5 SDI quintiles and 21 GBD regions in 2021. **(A)** The EAPC of ASIR for HSC in the reproductive age group. **(B)** The EAPC of ASIR for HSC in pre-elderly adults group. **(C)** The EAPC of ASIR for HSC in elderly adults group. EAPC, estimated annual percentage change; ASIR, age-standardized incidence rate; HSC, hormone-sensitive cancers; SDI, socio-demographic index; GBD, global burden of disease.

The ASIR in the Low SDI region increased from 12.04 (UI 9.48, 15.21) in 1990 to 19.13 (UI 15.57, 22.87) in 2021, while the ASIR in the High SDI region decreased from 55.62 (UI 53.56, 57.7) to 51.98 (UI 49.39, 54.58) over the same period (**Table 1**, **Figure 4A**). The EAPC for ASIR in the Low SDI region was 1.38 (CI 1.24, 1.52), indicating an upward trend, whereas the High SDI region showed a downward trend with an EAPC of -0.26 (CI -0.38, -0.14) (**Table 1**, **Figure 5A**). Similarly, ASDR and ASDiR followed identical trends between the Low and High SDI regions (**Table 1**; **Supplementary Figures 10A**, **11A**).

Among 21 GBD regions, developed regions in 2021 exhibited lower ASIR, ASDR, and ASDiR compared to underdeveloped regions, with corresponding EAPCs below 0, reflecting declining trends in incidence, mortality, and DALYs (**Supplementary Table 2**; **Figure 4A**). For example, Western Europe showed EAPCs for ASIR, ASDR, and ASDiR of -0.33 (CI -0.50, -0.15), -2.32 (CI -2.40, -2.25), and -2.14 (CI -2.21, -2.07), respectively (**Supplementary Table 2**, **Figure 5A**; **Supplementary Figures 12A**, **13A**). In contrast, underdeveloped African regions had EAPCs above 0 for all metrics, with North Africa and the Middle East and Southern Sub-Saharan Africa over 1.

#### 3.2.2 Regional burden and trend of HSC in PA group (50–74 years)

The ASIR in the Low SDI region rose from 71.86 (UI 56.77, 88.89) in 1990 to 102.77 (UI 85.84, 120.68) in 2021, while the High SDI region saw an increase from 335.87 (UI 319.8, 349.06) to 343.21 (UI 319.48, 361.52) (**Table 2**; **Figure 4B**). These incidence rates were significantly higher than those in the RA age group, with higher SDI countries exhibiting higher burden. However, the EAPC for ASIR in the High SDI region (0.12 [CI 0.02, 0.22]) was lower than that in the Low SDI region (1.11 [CI 0.97, 1.25]). In 2021, ASDR and ASDiR in the Low SDI region were 67 (UI 55.97, 78.33) and 1994.16 (UI 1666.79, 2334.01), respectively, compared to 69.09 (UI 64.63, 72.38) and 2184.49 (UI 2036.6, 2322.53) in the High SDI region. Although absolute values showed minimal differences, EAPCs of ASDR and ASDiR were below -1 in the High SDI region and above 0 in the Low SDI region (**Table 2**, **Supplementary Figures 10B**, **11B**).

Across 21 GBD regions, developed regions in 2021 had lower ASIR, ASDR, and ASDiR than underdeveloped regions. Except for High-income North America and Australasia, where EAPCs for ASIR were less than 0, most regions showed EAPCs greater than 0. EAPCs for ASDR and ASDiR were markedly lower in developed regions and inversely correlated with development levels. All African regions had EAPCs greater than 0 (**Supplementary Table 3**; **Figure 5B**; **Supplementary Figures 12B**, **13B**).

#### 3.2.3 Regional burden and trend of HSC in EA group (75+ years)

In 2021, ASIR increased progressively with higher SDI: 135.65 (UI 111.97, 158.61) in the Low SDI, 115.1 (UI 98.23, 134.87) in the Low-middle SDI, 136.04 (UI 114.06, 155.01) in the Middle SDI, 257.88 (UI 214.94, 286.6) in the High-middle SDI, and 465.27 (UI 376.7, 516.21) in the High SDI (**Table 3**). However, EAPCs for ASIR declined gradually: 1.44 (CI 1.30, 1.58) in the Low SDI, 1.62 (CI 1.54, 1.70) in the Low-middle SDI, 0.99 (CI 0.92, 1.06) in the Middle SDI, 0.86 (CI 0.73, 0.98) in the High-middle SDI and -0.03 (CI -0.09, 0.03) in the High SDI. Similar trends were observed for ASDR and ASDiR, with the High-middle and High SDI regions showing EAPCs less than 0 (**Table 3**; **Supplementary Figures 10C**, **11C**).

Among 21 GBD regions, developed regions in 2021 had higher ASIR, ASDR, and ASDiR but lower EAPCs compared to developing regions. All African regions exhibited EAPCs greater than 0, with North Africa and the Middle East reporting the highest EAPCs for ASIR, ASDR, and ASDiR: 4.36 (CI 4.01, 4.72), 2.67 (CI 2.37, 2.96), and 2.70 (CI 2.41, 3.00), respectively (**Supplementary Table 4**; **Figure 5C**; **Supplementary Figures 12C**, **13C**).

### 3.3 Inequity analysis

**Figure 6:**
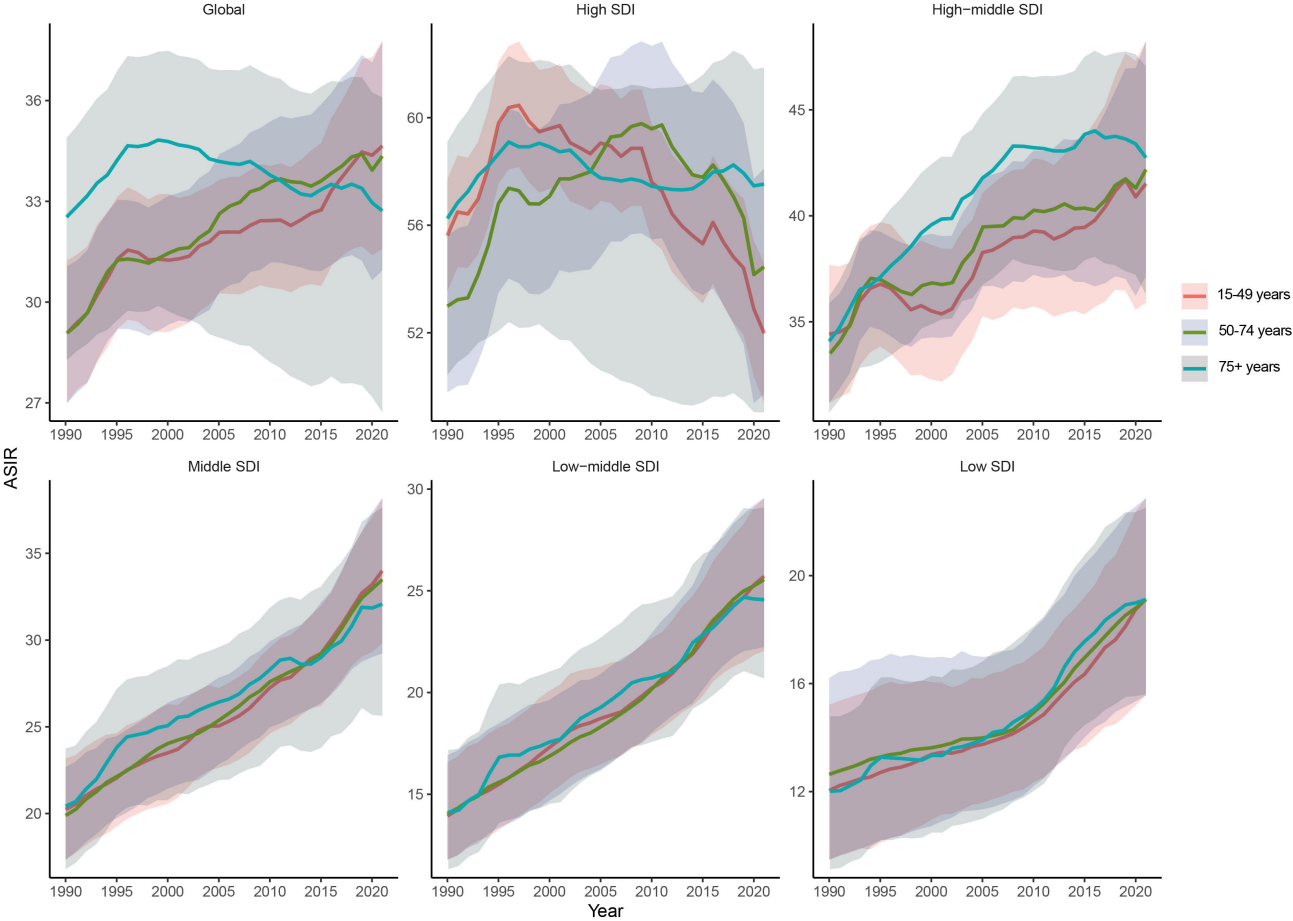
Time trend in ASIR of HSC among women of reproductive age, pre-elderly adults, and elderly adults across 5 SDI quintiles from 1990 to 2021. ASIR, age-standardized death rate; HSC, hormone-sensitive cancers; SDI, socio-demographic index.

**Figure 7:**
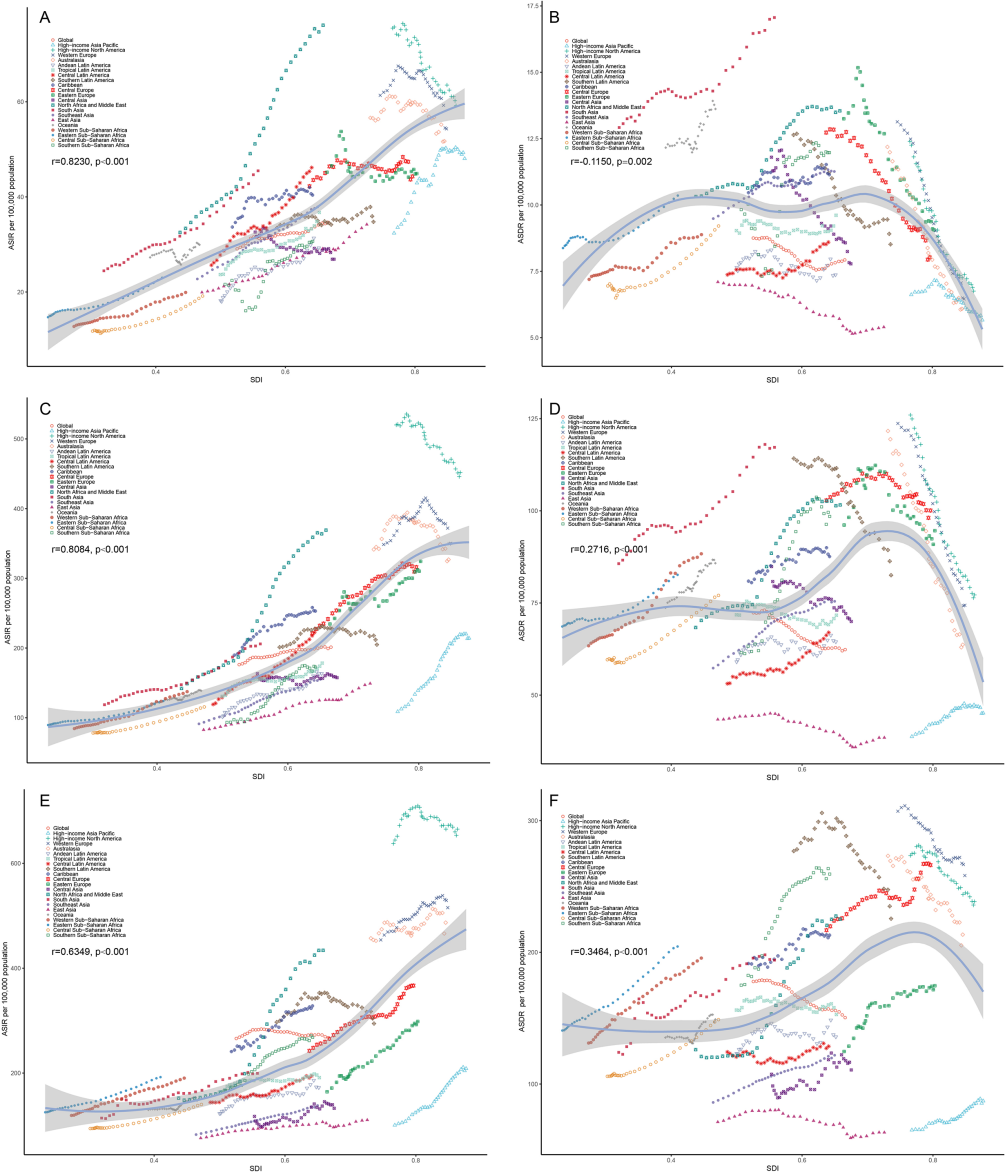
Spearman’s correlation between ASIR/ASDR for HSC and SDI among women of reproductive age, pre-elderly adults, and elderly adults across 21 GBD regions, 1990 to 2021. Each point represents the ASIR/ASDR corresponding to the SDI of the region in a given year, and the points are sequentially ordered from 1990 to 2021 from left to right. **(A, B)** The correlation of ASIR and ASDR with SDI among women of reproductive age. **(C, D)** The correlation of ASIR and ASDR with SDI among women of pre-elderly adults. **(E, F)** The correlation of ASIR and ASDR with SDI among women of elderly adults. ASIR, age-standardized incidence rate; ASDR, age-standardized death rate; HSC, hormone-sensitive cancers; SDI, socio-demographic index.

**Figure 8:**
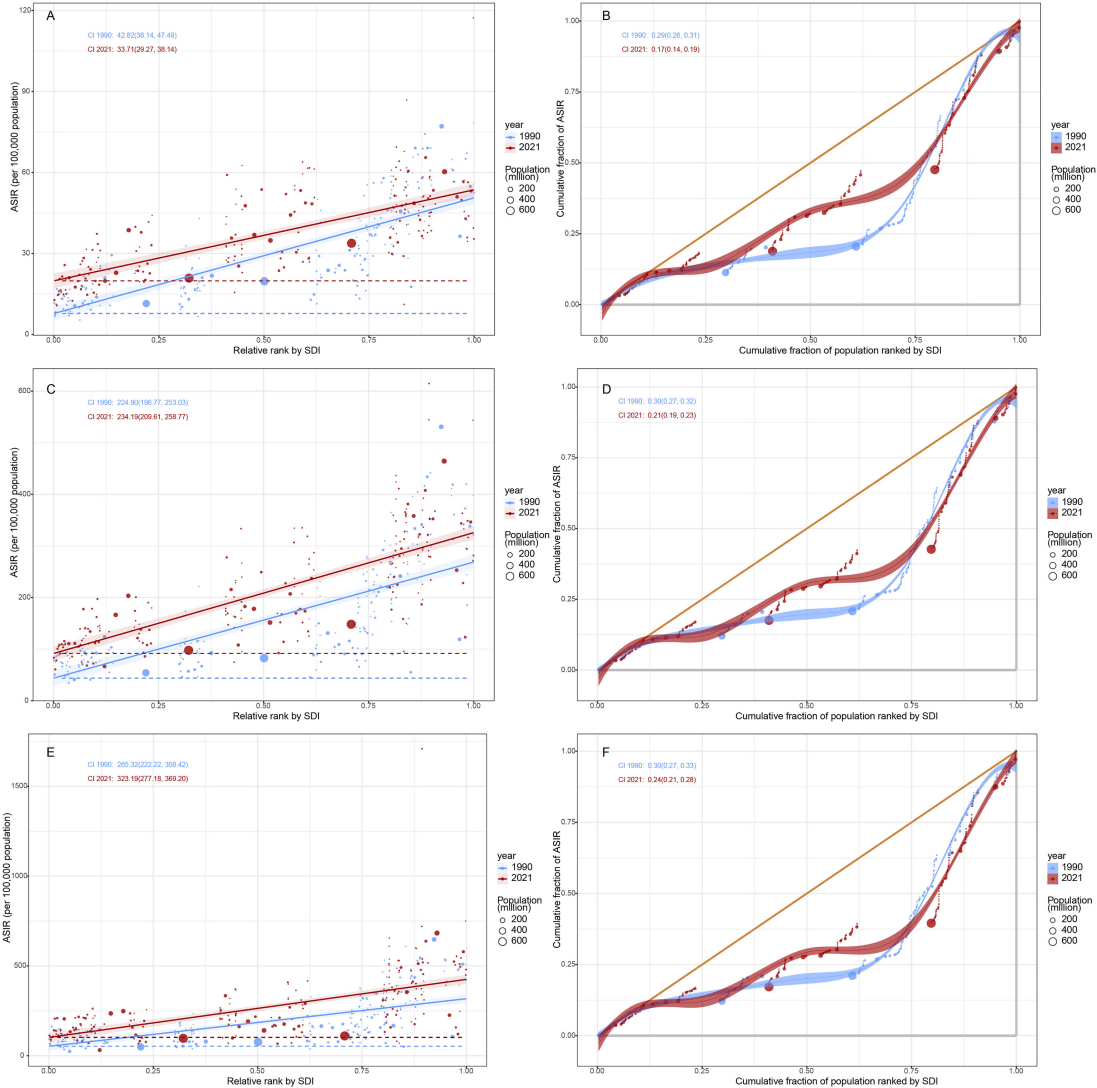
Inequality analysis of ASIR for HSC across all age groups in different countries/territories relative to their positions on the SDI scale, 1990 and 2021. **(A, C, E)** present the slope index of inequality for reproductive age, pre-elderly adults and elderly adults, respectively. **(B, D, F)** represent the concentration index for reproductive age, pre-elderly adults and elderly adults, respectively. ASIR, age-standardized incidence rate; HSC, hormone-sensitive cancers; SDI, socio-demographic index.

We analyzed the impact of different SDI levels on HSC. The incidence of HSCs, apart from decreasing in High SDI regions starting around 2010, showed an increasing trend over time in countries with other SDI levels (**Figure 6**). However, between 2019 and 2021, both the ASDR and ASDiR displayed a decreasing trend in the High and High-middle SDI regions, while other SDI regions showed an upward trend (**Supplementary Figures 14**, **S15**). The results revealed that in the PA age group, the ASIR increased with higher SDI (R = 0.8084, P<0.001, **Figure 7C**), while the ASDR (R = 0.2716, P<0.001, **Figure 7D**) and ASDiR (R = 0.3151, P<0.001, **Supplementary Figure 16B**) exhibited an initial rise followed by a decline. Similar trends were observed in the RA and EA age groups (**Figure 7**; **Supplementary Figure 16**). Further inequity analysis showed that as societies developed, inequality indices (slope and concentration index) for ASDR and ASDiR significantly decreased across all age groups (**Supplementary Figures 17**, **S18**). However, the slope index of inequality for ASIR widened in the PA and EA groups (from 224.90 to 234.19 and 265.32 to 323.19, respectively), while narrowing in the RA group (from 42.82 to 33.71) (**Figure 8**).

### 3.4 Decomposition analysis

**Figure 9:**
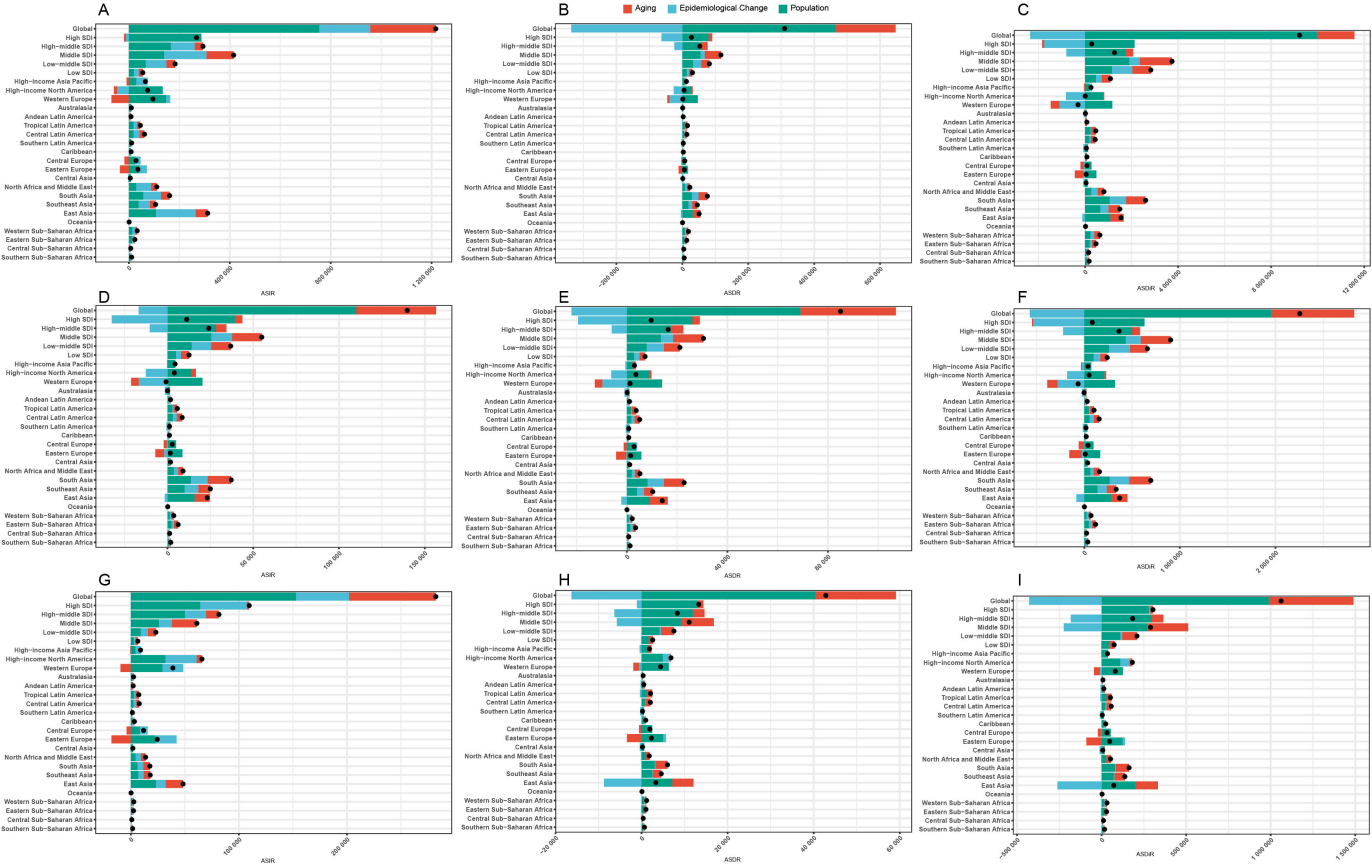
Decomposition analysis of changes in the ASIR/ASDR/ASDiR of HSC across all age groups from 1990 to 2021. **(A-C)** Decomposition analysis of changes in ASIR, ASDR, and ASDiR for the reproductive age, respectively. **(D-F)** Decomposition analysis of changes in ASIR, ASDR, and ASDiR for the pre-elderly adults, respectively. **(G-I)** Decomposition analysis of changes in ASIR, ASDR, and ASDiR for elderly adults, respectively. ASIR, age-standardized incidence rate; ASDR, age-standardized death rate; ASDiR, age-standardized disability-adjusted life years (DALYs) rate; HSC, hormone-sensitive cancers.

To assess the contributions of population growth, ageing, and epidemiological changes to HSC trends from 1990 to 2021, decomposition analyses were conducted for BC, OC, and UC For BC, population, ageing, and epidemiological changes accounted for 62.1%, 21.39%, and 16.5% of the total ASIR, respectively. In the High SDI region, ageing and epidemiological changes had a negative impact on BC incidence, contributing -2.94% and -4.25% respectively. Conversely, these factors had a positive influence on incidence in regions with lower SDI, with a contribution rate above 0. Notably, in developed regions such as Central Europe, Western Europe, Eastern Europe, High-income Asia Pacific, and High-income North America, ageing contributed negatively to incidence (-61.52%, -72.34%, -99.36%, -13.85%, and -17.07%, respectively) (**Figure 9A**).

For OC, population, ageing, and epidemiological changes contributed 78.73%, 33.31%, and -12.04% on ASIR, respectively. In the High and High-middle SDI region, epidemiological changes reduced OC burden (-290.88% and -42.88%), while population dominated 117.59% and 351.89%. The lower SDI region showed minimal variation in contributions from these factors (**Figure 9D**).

For UC, population, ageing, and epidemiological changes contributed 54.16%, 28.47%, and 17.37% on ASIR, respectively. Ageing had smaller contributions in the higher SDI regions (e.g., 14.81% in high-middle SDI and 2.06% in high SDI) compared to the lower SDI regions (29.11% in low SDI and 18.81% in low-middle SDI). In Eastern Europe, Central Europe, Western Europe, and the High-income Asia Pacific, ageing negatively contributed to UC incidence (-73.26%, -32.97%, -24.61%, and -4.76%). Epidemiological changes in the High SDI region reduced UC burden by -4.25% (**Figure 9G**).

ASDR and ASDiR followed similar patterns of ASIR across different age groups of HSCs. Specifically, in higher SDI regions, epidemiological change, and population factors accounted for a larger proportion of the ASDR and ASDiR, while aging had a higher contribution in lower SDI countries (**Figure 9**).

### 3.5 Frontier analysis

**Figure 10:**
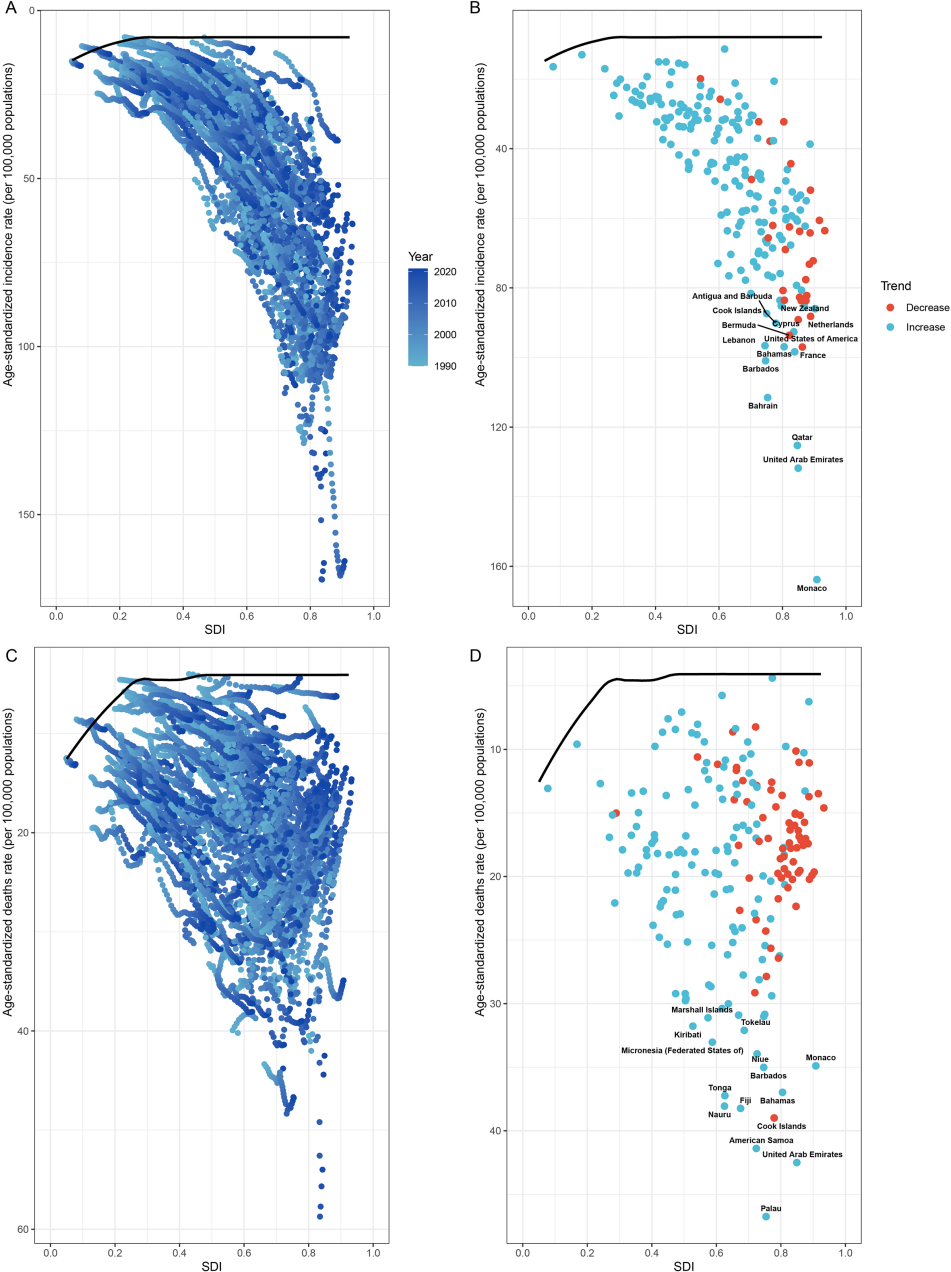
Frontier analysis based on SDI and ASIR/ASDR for breast cancer from 1990 to 2021. **(A, C)** represents the annual change in ASIR and ASDR for 204 countries/territories from 1990 to 2021, respectively, and the frontier line was generated in black solid. **(B, D)** each dot represents the difference between a specific country/territory and the frontier line in 2021. The decrease in the differences between a country/territory and the frontier line from 1990 to 2021 is marked in blue, while the increase is shown in red. SDI, socio-demographic index; ASIR, age-standardized incidence rate; ASDR, age-standardized death rate.

Frontier analysis was employed to examine the relationship between the burden of HSCs and SDI. The results revealed that from 1990 to 2021, countries/territories with varying levels of population development exhibited unrealized health gains, generally showing an expanding trend. Specifically, in 2021, countries/territories with different SDI levels demonstrated disparities in ASIR and ASDR for BC, along with effective differences (**Figure 10**). OC and UC showed similar patterns, but a smaller expanding trend was observed compared with BC (**Supplementary Figures 19**, **S20**). Higher SDI countries/territories had a larger gap between their ASIR and the frontier boundary, whereas the gap in ASDR was smaller. This indicates that countries/territories with different SDI levels have varying potential for improvement in different aspects of HSC epidemiology.

### 3.6 Risk factors attributable to HSC burden

**Figure 11:**
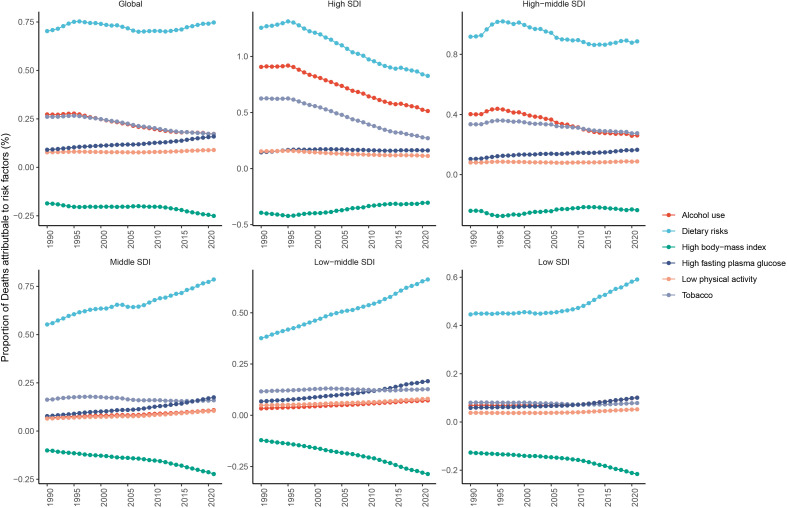
Trend of risk factors attributable to breast cancer ASDR among women of reproductive age across the 5 SDI quintiles from 1990 to 2021. ASDR, age-standardized death rate; SDI, socio-demographic index.

Risk factors for mortality in BC, OC, and UC were analyzed by age group from 1990 to 2021. For BC in the RA group, dietary risks, alcohol use, and tobacco were the top three contributors. These risks declined in the High and High-middle SDI regions but rose in the Middle, Low-middle, and Low SDI regions (**Figure 11**). In addition, high fasting plasma glucose emerged as a globally increasing risk factor in the RA age group. In the PA and EA groups, dietary risks, high body-mass index, and high fasting plasma glucose were predominant risk factors, with similar declining trends in the High SDI region and rising trends in the lower SDI regions (**Supplementary Figure 21**).

For OC, the high body-mass index declined as a risk factor in the RA group globally but increased in the Middle, Low-middle, and Low SDI regions. In the PA and EA groups, the high body-mass index stabilized in the High and High-middle SDI regions but rose in the lower SDI regions (**Supplementary Figure 22**).

For UC, the high body-mass index showed an increasing impact across all ages, with linear upward trends in the Middle, Low-Middle, and Low SDI regions, and fluctuating increases were observed in the High and High-middle SDI regions (**Supplementary Figure 23**).

### 3.7 Trend forecast to 2035

**Figure 12:**
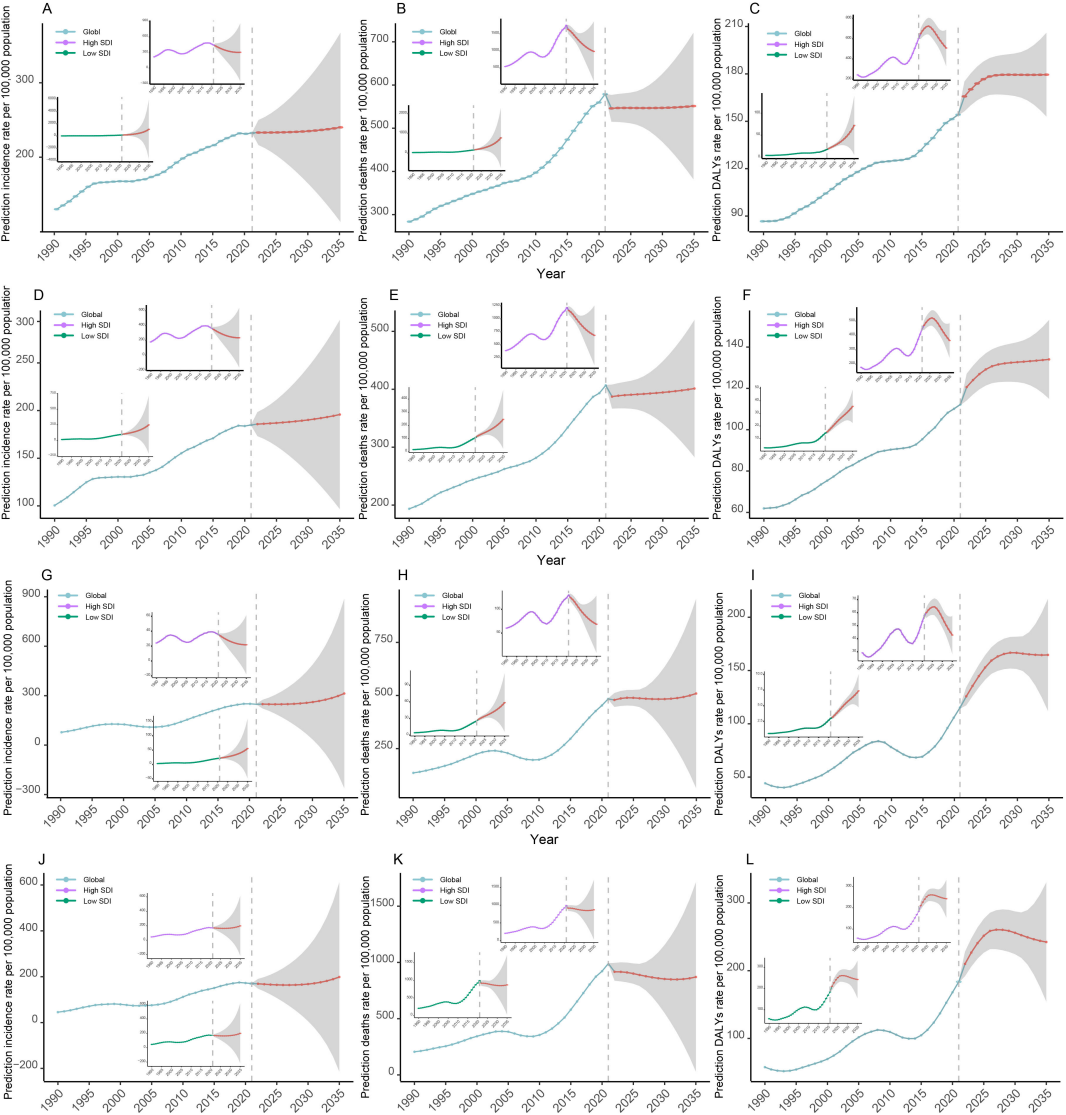
Projection of HSC incidence, mortality and DALYs rate trends through 2035. **(A-C)** Projected trends in the overall incidence, mortality, and DALY rates for HSCs, respectively. **(D-F)** Projected trends in the incidence, mortality, and DALYs rates for breast cancer, respectively. **(G-I)** Projected trends in the incidence, mortality, and DALYs rates for ovarian cancer, respectively. **(J-L)** Projected trends in the incidence, mortality, and DALYs rates for uterine cancer, respectively. HSC, hormone-sensitive cancers; DALYs, age-standardized disability-adjusted life years.

Using the BAPC model, we projected HSC incidence trends to 2035. Global HSC incidence is expected to rise slowly in the RA group, stabilize in the PA group, and increase before stabilizing in the EA group. Predictions for the Low SDI region indicate continued increases across all age groups, whereas the High SDI region may see declines (**Figures 12A–C**). Similar patterns were observed for BC, OC, and UC (**Figures 12D–L**).

## 4 Discussion

This is the first study to provide a comprehensive assessment of the global burden, trends, and inequalities in hormone-sensitive cancers among women aged 15 years and older from 1990 to 2021, with projections to 2035. In 2021, BC remained the most prevalent HSC, with an ASIR of 28.81 (UI 26.84, 30.94) out of a total of 34.65 (UI 31.59, 37.76). Globally, from 1990 to 2021, the reproductive age group exhibits the lowest values in ASIR, ASDR, and ASDiR, all showing an upward trend. Conversely, the elderly adult group has the highest values in these metrics, with a rapid decline observed over the same period. Across all age groups, ASIR values are higher in regions with higher SDI levels. ASDR and ASDiR, however, initially rise and then decline as SDI increases. Obesity-related risk factors are the primary contributors to HSC-related mortality. The BAPC projections indicate that these trends are expected to continue through 2035.

The HSCs account for approximately 30% to 40% of all female cancers, making their prevalence and disease burden significant global public health concerns (26, 27) In 2017, HSCs were responsible for a macroeconomic loss of $2.676 trillion worldwide, and projections indicated that these losses will reach 0.058% of the global GDP between 2020 and 2050 (28). Additionally, as the ageing of the global female population and changes in lifestyle and fertility patterns, the burden of HSCs continues to increase, posing a substantial challenge to public health (5, 29, 30). As a result, there have been notable changes in the incidence patterns of HSCs among women across different age groups and regions. Therefore, in-depth research is urgently needed to develop targeted health strategies to mitigate the impact of these cancers on women’s health worldwide.

Age stratification plays a crucial role in shaping the epidemiological characteristics of HSCs. The onset of these diseases exhibits a distinct age-dependent pattern, with hormone levels undergoing significant changes during ageing (3). Globally, BC and OC incidence peaks in the 55–59 age group, while UC reaches its highest rate in the 60–64 age group (27). In 2015, high-risk age groups for BC varied across regions: 65–69 years in China, 50–54 years in India, and 60–64 years in Thailand, likely influenced by dietary habits, lifestyle, and cultural factors (31). Furthermore, global population ageing has exacerbated the burden of BC. In Europe, where over 20% of the population is aged 60+, incidence rates among the elderly have risen sharply. Similarly, ageing populations in Asia, driven by increased life expectancy and reduced fertility rate, face growing disease burdens (32). In elderly patients, declining physical function may increase the risk of drug-related adverse reactions or complications, further complicating treatment and resource utilization (33, 34). However, the trend of delay in childbearing age has led to a significant increase in incidence rates among women of reproductive age (35, 36). For example, Asian countries exhibit earlier peak incidence ages (e.g., 40–59 in China, 45–49 in South Korea) compared to Western nations, potentially linked to delayed childbirth, declining fertility rates, and altered hormone exposure patterns (32). Our analysis also shows that women in the 75+ age group have the highest absolute incidence of HSCs across all age groups, although the growth rate has been declining. In contrast, the 15–49 age group, while having a lower incidence rate, is experiencing the fastest growth. Therefore, understanding the age stratification of HSCs is crucial for developing targeted healthcare strategies that optimize resource allocation and improve the management of comorbidities, ultimately enhancing the quality of care for affected individuals.

Significant regional variations in HSC epidemiology are largely driven by a complex interplay between economic development, healthcare accessibility, and structural gender inequalities. Our study reveals that while high-SDI regions maintain higher baseline ASIR due to advanced diagnostic saturation and Westernized lifestyle risks, their EAPCs show gradual declines, reflecting stabilized healthcare systems and improved management of mortality (ASDR and ASDiR). Conversely, low-SDI regions face a paradoxical “low baseline, high growth” pattern. These disparities are not merely economic but are deeply intertwined with gender-based structural vulnerabilities (13, 37). In lower SDI settings, women often navigate a “double-burden” of health inequity: biological risk factors are compounded by restricted autonomy in healthcare decision-making and systemic underinvestment in female-specific diagnostic infrastructure (38, 39). This explains why these regions exhibit the most rapid escalation in HSC burden despite lower initial incidence rates. As the rate of increase in mortality and DALYs continues to rise in low-SDI regions while slowing in high-SDI areas. This persistent healthcare divide remains a critical barrier, highlighted by the intersection of gender and socioeconomic status (13). Without targeted interventions to address these gender-responsive structural gaps, the escalating HSC burden in developing nations will likely hinder their economic development and exacerbate global health inequities in the foreseeable future.

Western diets are typically characterized by high protein and fat, in contrast to the high-fiber, low-fat diets prevalent in many developing regions (40). However, as lower SDI countries undergo industrialization, their dietary habits are converging with Western norms (41), which may correlate with rising incidence and mortality rates of HSCs. Notably, inequality analyses reveal widening gaps in incidence between High- and Low-SDI regions, while mortality and DALYs gaps are narrowing slightly. This paradoxical trend may reflect accelerated population ageing in the High SDI countries, leading to increased absolute mortality and DALYs, thereby reducing the disparity with Low SDI regions (42, 43). Frontier analyses further indicate that higher SDI regions exhibit larger gaps from the optimal ASIR frontier line but smaller gaps in ASDR. Projections to 2035 suggest that the incidence of HSCs will continue to rise in lower SDI regions. Thus, despite narrowing mortality gaps, the persistent healthcare divide between developed and developing nations demands attention, as evidenced by the 13-times difference in ASIR between Niger and Monaco observed in our study.

Obesity-related factors, including dietary risks, high body mass index, and high fasting plasma glucose, account for the majority of HSC burdens (44). These risk factors attributed to mortality and DALYs show divergent trends across SDI regions: they are rising in Low SDI regions but declining in High SDI regions. This emphasizes the impact of socioeconomic development, urbanization, and lifestyle changes on mortality patterns, highlighting the need for targeted interventions in developing countries (45, 47). To avoid replicating the adverse epidemiological trajectories observed in the High SDI regions, Low SDI regions must implement proactive public health strategies, prioritize health education, and mitigate economic losses caused by HSCs (48, 49).

Given that the escalating burden among women of reproductive age threatens to exacerbate macroeconomic productivity losses and deepen global health inequities, it is imperative to translate these epidemiological insights into actionable public health frameworks. To this end, several gender-responsive policy pillars are proposed. First, targeted resource reallocation for high-EAPC regions: Countries in the Middle East and Africa with EAPCs, where growth rates are most aggressive, must transition from opportunistic to systematic screening. These programs should specifically prioritize the reproductive age group to mitigate the loss of the functional labor force. Second, narrowing the “Frontier Gap” through technological leapfrogging: Low-SDI nations should bypass traditional, capital-intensive infrastructure in favor of cost-effective, mobile-based diagnostic tools and decentralized oncology networks. Third, integrating metabolic and reproductive health services: Since obesity-related factors are primary drivers of HSC mortality, national metabolic health programs should be embedded within maternal and reproductive health platforms. This integration would foster a life-course approach to HSC prevention, thereby avoiding the adverse epidemiological trajectories previously observed in high-SDI regions.

This study has limitations: (1) While we use the term “hormone-sensitive”, this epidemiological analysis does not distinguish between molecular subtypes due to GBD data constraints. We acknowledge that not all cases are classically hormone-dependent; (2) Reliance on the GBD database may introduce biases due to variations in data collection methods and data acquisition technology across countries/territories, potentially affecting completeness and accuracy; (3) Underdiagnosis and underreporting in the lower SDI regions may lead to underestimated incidence, deaths and DALYs rates; (4) The impact of the COVID-19 pandemic (2019–2021) warrants a cautious interpretation of the most recent trends in our Frontier and Decomposition analyses. During this period, global redirections of healthcare resources and disruptions in elective oncology screenings likely led to transient underreporting or delayed diagnoses. Nevertheless, this research offers critical insights: (1) This study investigated the trend of HSC in 204 countries and regions in the past 30 years, and predicted its change in the next 14 years, which helps understand the relationship between the epidemiological characteristics of HSCs and social development in different countries/territories. It may provide references for each country to formulate personalized medical and health strategies for HSC; (2) By highlighting global inequalities, it informs the equitable allocation of healthcare resources and advances global health equity.

## 5 Conclusion

By analyzing the GBD data from 1990 to 2021, we observed that the burden of HSCs was stratified by different age groups, and BC remained the most prevalent HSC. Our analysis also revealed significant inequalities in HSC incidence and mortality across different SDI levels. These global trends are projected to continue over the next decade. This study provides a basis for formulating relevant policies and allocating limited resources effectively.

## Data Availability

The original contributions presented in the study are included in the article/**Supplementary Material**. Further inquiries can be directed to the corresponding author.

## Supplementary material

Supplementary Figure 1 Global distribution of EAPC of ASIR for breast cancer among women of reproductive age, pre-elderly adults, and elderly adults in 2021. **(A)** The EAPC of ASIR for breast cancer in the reproductive age group. **(B)** The EAPC of ASIR for breast cancer in pre-elderly adults group. **(C)** The EAPC of ASIR for breast cancer in elderly adults group. EAPC, estimated annual percentage change; ASIR, age-standardized incidence rate; HSC, hormone-sensitive cancers.

Supplementary Figure 2 Global distribution of EAPC of ASIR for uterine cancer among women of reproductive age, pre-elderly adults, and elderly adults in 2021. **(A)** The EAPC of ASIR for uterine cancer in the reproductive age group. **(B)** The EAPC of ASIR for uterine cancer in pre-elderly adults group. **(C)** The EAPC of ASIR for uterine cancer in elderly adults group. EAPC, estimated annual percentage change; ASIR, age-standardized incidence rate; HSC, hormone-sensitive cancers.

Supplementary Figure 3 Global distribution of EAPC of ASIR for ovarian cancer among women of reproductive age, pre-elderly adults, and elderly adults in 2021. **(A)** The EAPC of ASIR for ovarian cancer in the reproductive age group. **(B)** The EAPC of ASIR for ovarian cancer in pre-elderly adults group. **(C)** The EAPC of ASIR for ovarian cancer in elderly adults group. EAPC, estimated annual percentage change; ASIR, age-standardized incidence rate; HSC, hormone-sensitive cancers.

Supplementary Figure 4 Global distribution of EAPC of ASDR for breast cancer among women of reproductive age, pre-elderly adults, and elderly adults in 2021. **(A)** The EAPC of ASDR for breast cancer in the reproductive age group. **(B)** The EAPC of ASDR for breast cancer in pre-elderly adults group. **(C)** The EAPC of ASDR for breast cancer in elderly adults group. EAPC, estimated annual percentage change; ASIR, age-standardized death rate; HSC, hormone-sensitive cancers.

Supplementary Figure 5 Global distribution of EAPC of ASDR for uterine cancer among women of reproductive age, pre-elderly adults, and elderly adults in 2021. **(A)** The EAPC of ASDR for uterine cancer in the reproductive age group. **(B)** The EAPC of ASDR for uterine cancer in pre-elderly adults group. **(C)** The EAPC of ASDR for uterine cancer in elderly adults group. EAPC, estimated annual percentage change; ASIR, age-standardized death rate; HSC, hormone-sensitive cancers.

Supplementary Figure 6 Global distribution of EAPC of ASDR for ovarian cancer among women of reproductive age, pre-elderly adults, and elderly adults in 2021. **(A)** The EAPC of ASDR for ovarian cancer in the reproductive age group. **(B)** The EAPC of ASDR for ovarian cancer in pre-elderly adults group. **(C)** The EAPC of ASDR for ovarian cancer in elderly adults group. EAPC, estimated annual percentage change; ASIR, age-standardized death rate; HSC, hormone-sensitive cancers.

Supplementary Figure 7 Global distribution of EAPC of ASDiR for breast cancer among women of reproductive age, pre-elderly adults, and elderly adults in 2021. **(A)** The EAPC of ASDiR for breast cancer in the reproductive age group. **(B)** The EAPC of ASDiR for breast cancer in pre-elderly adults group. **(C)** The EAPC of ASDiR for breast cancer in elderly adults group. EAPC, estimated annual percentage change; ASDiR, age-standardized disability-adjusted life years (DALYs) rate; HSC, hormone-sensitive cancers.

Supplementary Figure 8 Global distribution of EAPC of ASDiR for uterine cancer among women of reproductive age, pre-elderly adults, and elderly adults in 2021. **(A)** The EAPC of ASDiR for uterine cancer in the reproductive age group. **(B)** The EAPC of ASDiR for uterine cancer in pre-elderly adults group. **(C)** The EAPC of ASDiR for uterine cancer in elderly adults group. EAPC, estimated annual percentage change; ASDiR, age-standardized disability-adjusted life years (DALYs) rate; HSC, hormone-sensitive cancers.

Supplementary Figure 9 Global distribution of EAPC of ASDiR for ovarian cancer among women of reproductive age, pre-elderly adults, and elderly adults in 2021. **(A)** The EAPC of ASDiR for ovarian cancer in the reproductive age group. **(B)** The EAPC of ASDiR for ovarian cancer in pre-elderly adults group. **(C)** The EAPC of ASDiR for ovarian cancer in elderly adults group. EAPC, estimated annual percentage change; ASDiR, age-standardized disability-adjusted life years (DALYs) rate; HSC, hormone-sensitive cancers.

Supplementary Figure 10 Regional ASDR barplot for HSC among women of reproductive age, pre-elderly adults, and elderly adults in 1990, 2010, and 2021. **(A)** The ASDR for HSC in the reproductive age group. **(B)** The ASDR for HSC in pre-elderly adults group. **(C)** The ASDR for HSC in elderly adults group. ASDR, age-standardized death rate; HSC, hormone-sensitive cancers.

Supplementary Figure 11 The EAPC of ASDR for HSC among women of reproductive age, pre-elderly adults, and elderly adults across 5 SDI quintiles and 21 GBD regions in 2021. **(A)** The EAPC of ASDR for HSC in the reproductive age group. **(B)** The EAPC of ASDR for HSC in pre-elderly adults group. **(C)** The EAPC of ASDR for HSC in elderly adults group. EAPC, estimated annual percentage change; ASDR, age-standardized death rate; HSC, hormone-sensitive cancers; SDI, socio-demographic index; GBD, global burden of disease.

Supplementary Figure 12 Regional ASDiR barplot for HSC among women of reproductive age, pre-elderly adults, and elderly adults in 1990, 2010, and 2021. **(A)**. The ASDiR for HSC in the reproductive age group. **(B)**. The ASDiR for HSC in pre-elderly adults group. **(C)**. The ASDiR for HSC in elderly adults group. ASDiR, age-standardized disability-adjusted life years (DALYs) rate; HSC, hormone-sensitive cancers.

Supplementary Figure 13 The EAPC of ASDiR for HSC among women of reproductive age, pre-elderly adults, and elderly adults across 5 SDI quintiles and 21 GBD regions in 2021. **(A)**. The EAPC of ASDiR for HSC in the reproductive age group. **(B)**. The EAPC of ASDiR for HSC in pre-elderly adults group. **(C)**. The EAPC of ASDiR for HSC in elderly adults group. EAPC, estimated annual percentage change; ASDiR, age-standardized disability-adjusted life years (DALYs) rate; SDI, socio-demographic index; GBD, global burden of disease.

Supplementary Figure 14 Time trend in ASDR of HSC among women of reproductive age, pre-elderly adults, and elderly adults across 5 SDI quintiles from 1990 to 2021. ASDR, age-standardized death rate; HSC, hormone-sensitive cancers; SDI, socio-demographic index.

Supplementary Figure 15 Time trend in ASDiR of HSC among women of reproductive age, pre-elderly adults, and elderly adults across 5 SDI quintiles from 1990 to 2021. ASDiR, age-standardized disability-adjusted life years (DALYs) rate; HSC, hormone-sensitive cancers; SDI, socio-demographic index.

Supplementary Figure 16 Spearman’s correlation between ASDiR for HSC and SDI among women of reproductive age, pre-elderly adults, and elderly adults across 21 GBD regions, 1990 to 2021. Each point represents the ASDiR corresponding to the SDI of the region in a given year, and the points are sequentially ordered from 1990 to 2021 from left to right. **(A)**, The correlation of ASDiR with SDI among women of reproductive age. **(B)**, The correlation of ASDiR with SDI among women of pre-elderly adults. **(C)**, The correlation of ASDiR with SDI among women of elderly adults. ASDiR, age-standardized disability-adjusted life years (DALYs) rate; HSC, hormone-sensitive cancers; SDI, socio-demographic index.

Supplementary Figure 17 Inequality analysis of ASDR for HSC across all age groups in different countries/territories relative to their positions on the SDI scale, 1990 and 2021. **(A, C, E)** present the slope index of inequality for reproductive age, pre-elderly adults and elderly adults, respectively. **(B, D, F)** represent the concentration index for reproductive age, pre-elderly adults and elderly adults, respectively. ASDR, age-standardized death rate; HSC, hormone-sensitive cancers; SDI, socio-demographic index.

Supplementary Figure 18 Inequality analysis of ASDiR for HSC across all age groups in different countries/territories relative to their positions on the SDI scale, 1990 and 2021. **(A, C, E)** present the slope index of inequality for reproductive age, pre-elderly adults and elderly adults, respectively. **(B, D, F)** represent the concentration index for reproductive age, pre-elderly adults and elderly adults, respectively. ASDiR, age-standardized disability-adjusted life years (DALYs) rate; HSC, hormone-sensitive cancers; SDI, socio-demographic index.

Supplementary Figure 19 Frontier analysis based on SDI and ASIR/ASDR for ovarian cancer from 1990 to 2021. **(A, C)**: represents the annual change in ASIR and ASDR for 204 countries/territories from 1990 to 2021, respectively, and the frontier line was generated in black solid. **(B, D)**: each dot represents the difference between a specific country/territory and the frontier line in 2021. The decrease in the differences between a country/territory and the frontier line from 1990 to 2021 is marked in blue, while the increase is shown in red. SDI, socio-demographic index; ASIR, age-standardized incidence rate; ASDR, age-standardized death rate.

Supplementary Figure 20 Frontier analysis based on SDI and ASIR/ASDR for uterine cancer from 1990 to 2021. **(A, C)**: represents the annual change in ASIR and ASDR for 204 countries/territories from 1990 to 2021, respectively, and the frontier line was generated in black solid. **(B, D)**: each dot represents the difference between a specific country/territory and the frontier line in 2021. The decrease in the differences between a country/territory and the frontier line from 1990 to 2021 is marked in blue, while the increase is shown in red. SDI, socio-demographic index; ASIR, age-standardized incidence rate; ASDR, age-standardized death rate.

Supplementary Figure 21 Trend of risk factors attributable to breast cancer ASDR among women of pre-elderly adults and elderly adults across the 5 SDI quintiles from 1990 to 2021. ASDR, age-standardized death rate; SDI, socio-demographic index.

Supplementary Figure 22 Trend of risk factors attributable to ovarian cancer ASDR among women of reproductive age, pre-elderly adults, and elderly adults age across the 5 SDI quintiles from 1990 to 2021. ASDR, age-standardized death rate; SDI, socio-demographic index.

Supplementary Figure 23 Trend of risk factors attributable to uterine cancer ASDR among women of reproductive age, pre-elderly adults, and elderly adults across the 5 SDI quintiles from 1990 to 2021. ASDR, age-standardized death rate; SDI, socio-demographic index.
