# Supplementary material for: Future landscapes of women’s hormone-sensitive cancers: unraveling global trends, age stratification, and regional inequities (1990–2035)
Source: Front Endocrinol (Lausanne). 2026 May 1;17:1691889. doi: 10.3389/fendo.2026.1691889 (PMC13177307; doi:10.3389/fendo.2026.1691889)
Supplement: Supplementary Figure 1 — Global distribution of EAPC of ASIR for breast cancer among women of reproductive age, pre-elderly adults, and elderly adults in 2021. (A) The EAPC of ASIR for breast cancer in the reproductive age group. (B) The EAPC of ASIR for breast cancer in pre-elderly adults group. (C) The EAPC of ASIR for breast cancer in elderly adults group. EAPC, estimated annual percentage change; ASIR, age-standardized incidence rate; HSC, hormone-sensitive cancers. [file DataSheet1.docx]

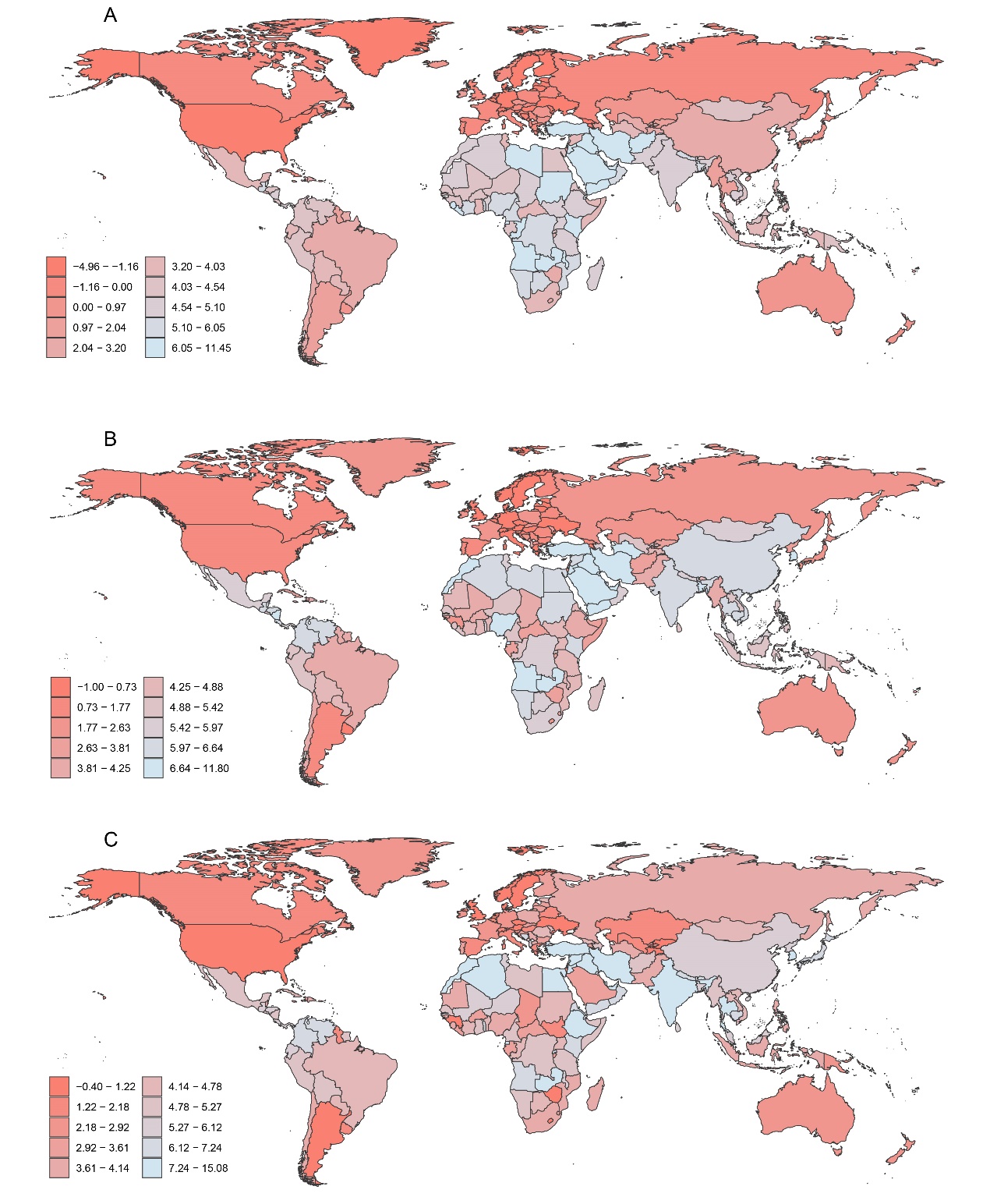


Figure S1 Global distribution of EAPC of ASIR for breast cancer among women of reproductive age, pre-elderly adults, and elderly adults in 2021.

A. The EAPC of ASIR for breast cancer in the reproductive age group. B. The EAPC of ASIR for breast cancer in pre-elderly adults group. C. The EAPC of ASIR for breast cancer in elderly adults group. EAPC: estimated annual percentage change; ASIR: age-standardized incidence rate; HSC: hormone-sensitive cancers.


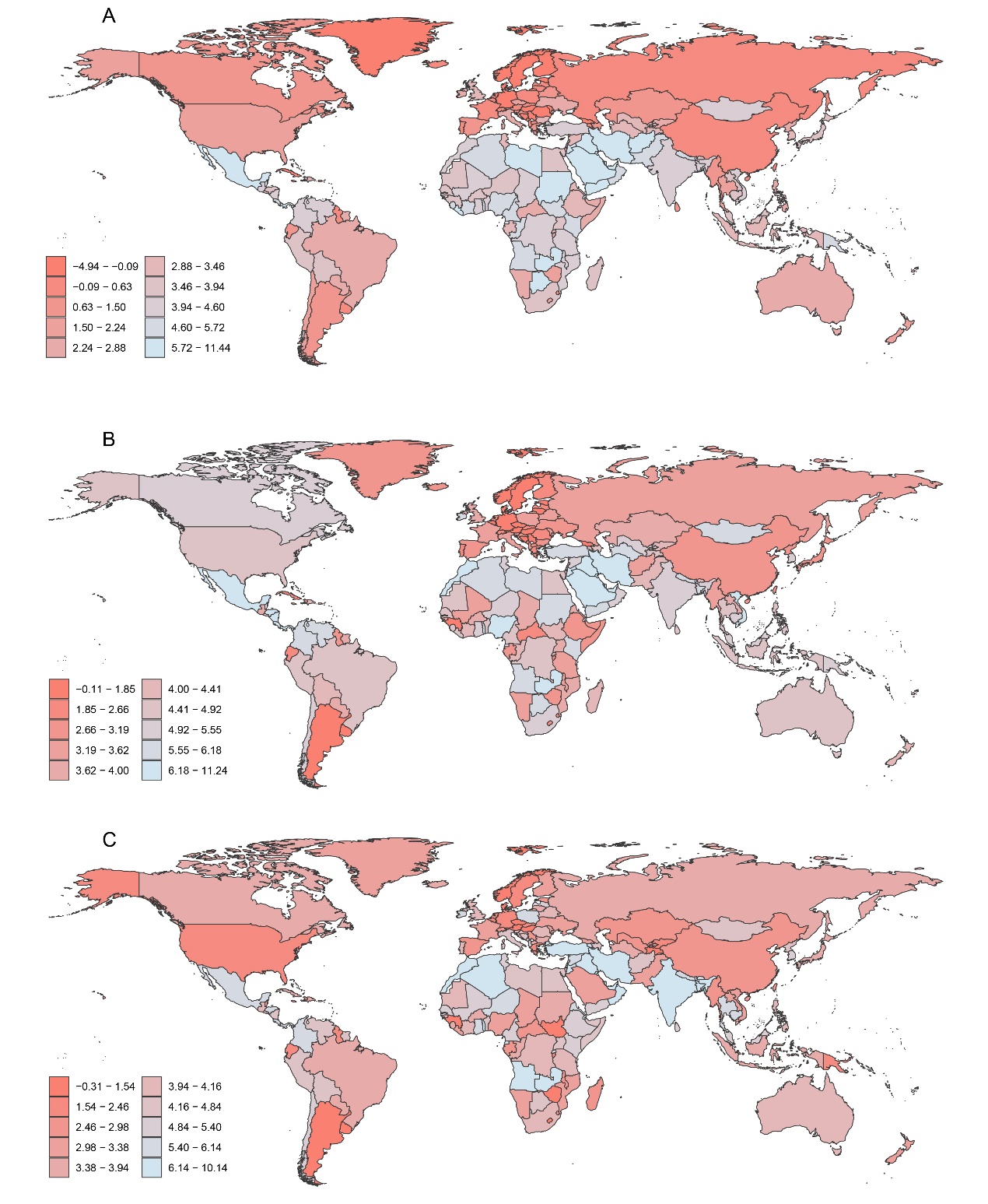


Figure S2 Global distribution of EAPC of ASIR for uterine cancer among women of reproductive age, pre-elderly adults, and elderly adults in 2021.

A. The EAPC of ASIR for uterine cancer in the reproductive age group. B. The EAPC of ASIR for uterine cancer in pre-elderly adults group. C. The EAPC of ASIR for uterine cancer in elderly adults group. EAPC: estimated annual percentage change; ASIR: age-standardized incidence rate; HSC: hormone-sensitive cancers.


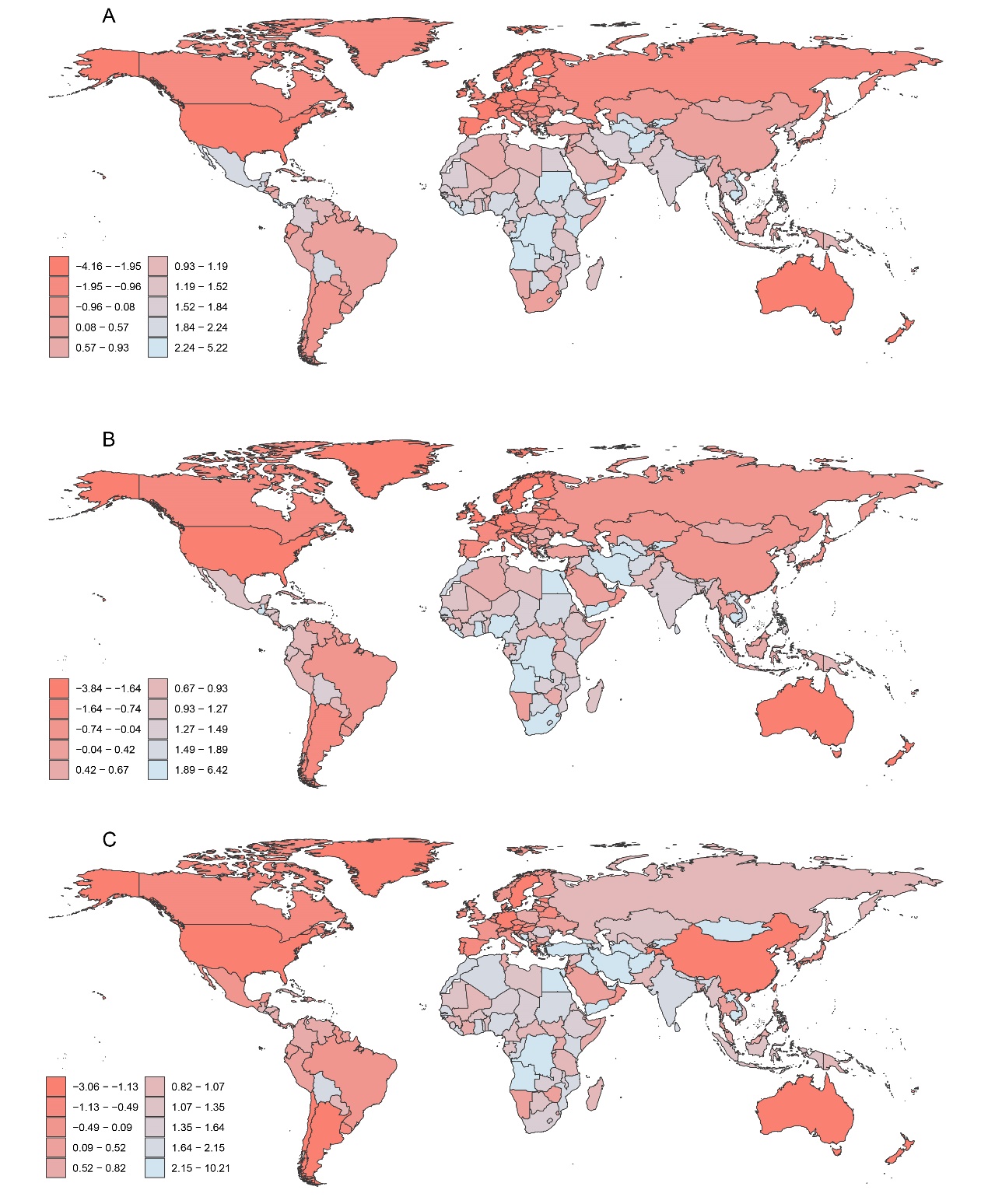


Figure S3 Global distribution of EAPC of ASIR for ovarian cancer among women of reproductive age, pre-elderly adults, and elderly adults in 2021.

A. The EAPC of ASIR for ovarian cancer in the reproductive age group. B. The EAPC of ASIR for ovarian cancer in pre-elderly adults group. C. The EAPC of ASIR for ovarian cancer in elderly adults group. EAPC: estimated annual percentage change; ASIR: age-standardized incidence rate; HSC: hormone-sensitive cancers.


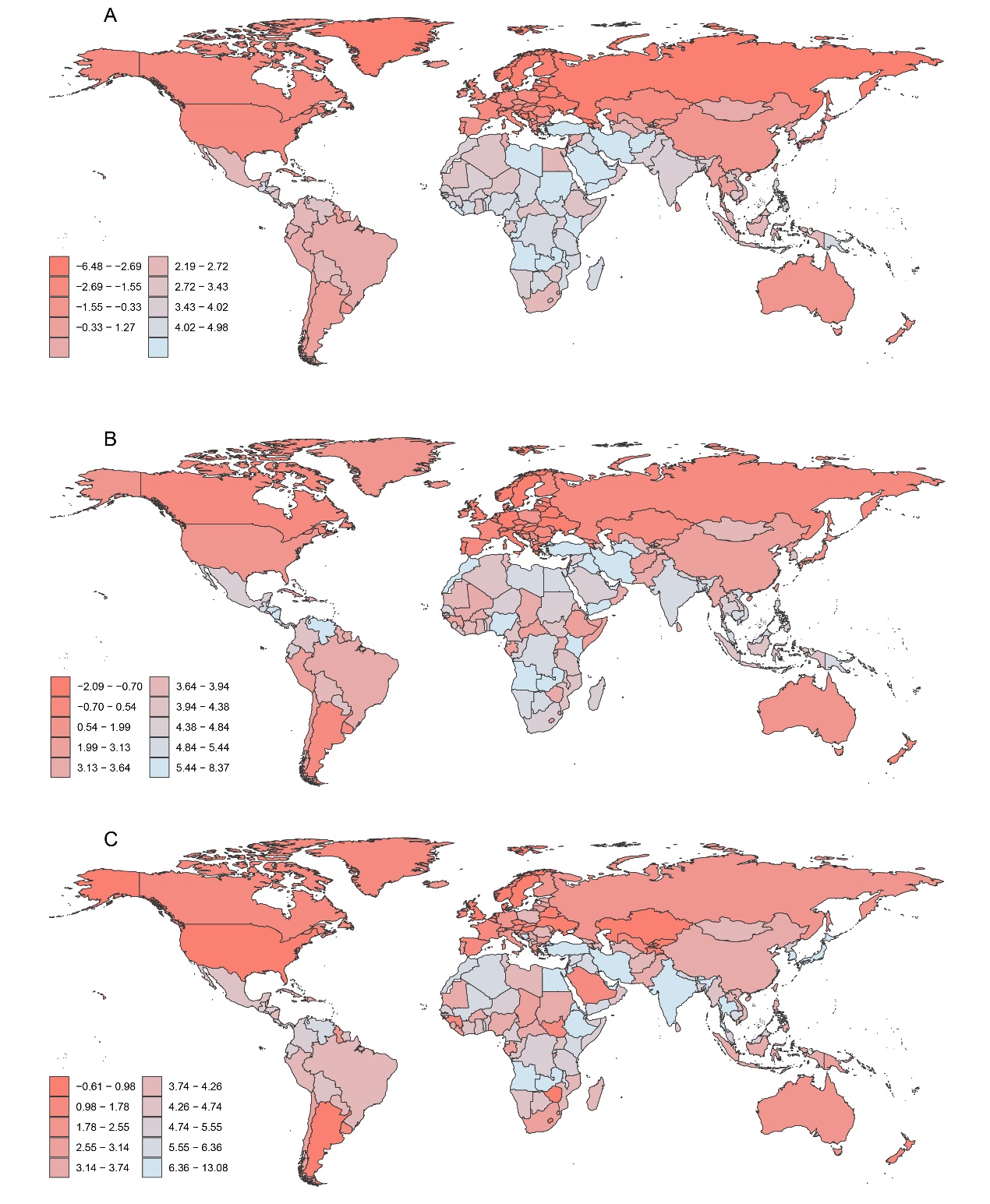


Figure S4 Global distribution of EAPC of ASDR for breast cancer among women of reproductive age, pre-elderly adults, and elderly adults in 2021.

A. The EAPC of ASDR for breast cancer in the reproductive age group. B. The EAPC of ASDR for breast cancer in pre-elderly adults group. C. The EAPC of ASDR for breast cancer in elderly adults group. EAPC: estimated annual percentage change; ASIR: age-standardized death rate; HSC: hormone-sensitive cancers.


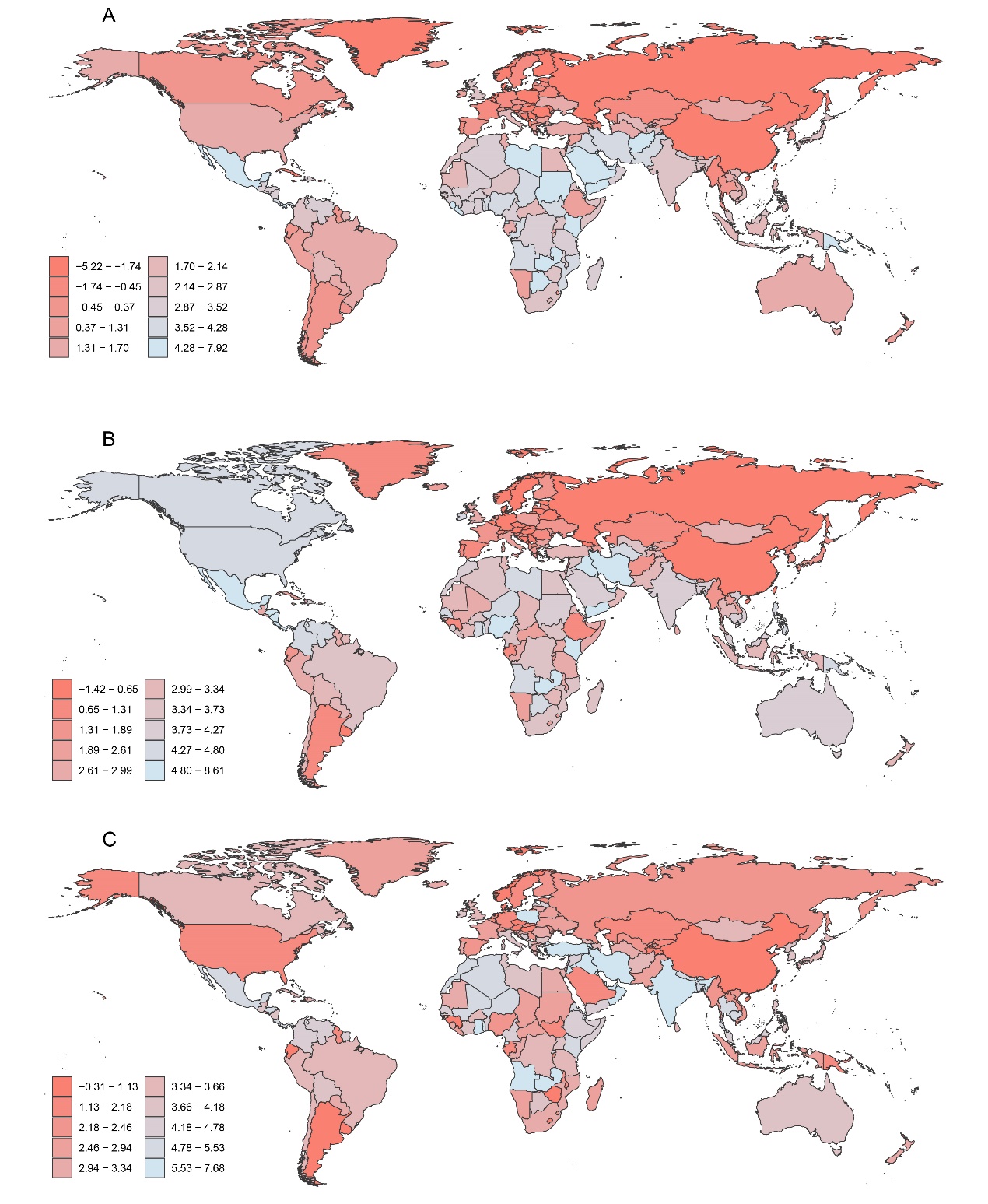


Figure S5 Global distribution of EAPC of ASDR for uterine cancer among women of reproductive age, pre-elderly adults, and elderly adults in 2021.

A. The EAPC of ASDR for uterine cancer in the reproductive age group. B. The EAPC of ASDR for uterine cancer in pre-elderly adults group. C. The EAPC of ASDR for uterine cancer in elderly adults group. EAPC: estimated annual percentage change; ASIR: age-standardized death rate; HSC: hormone-sensitive cancers.


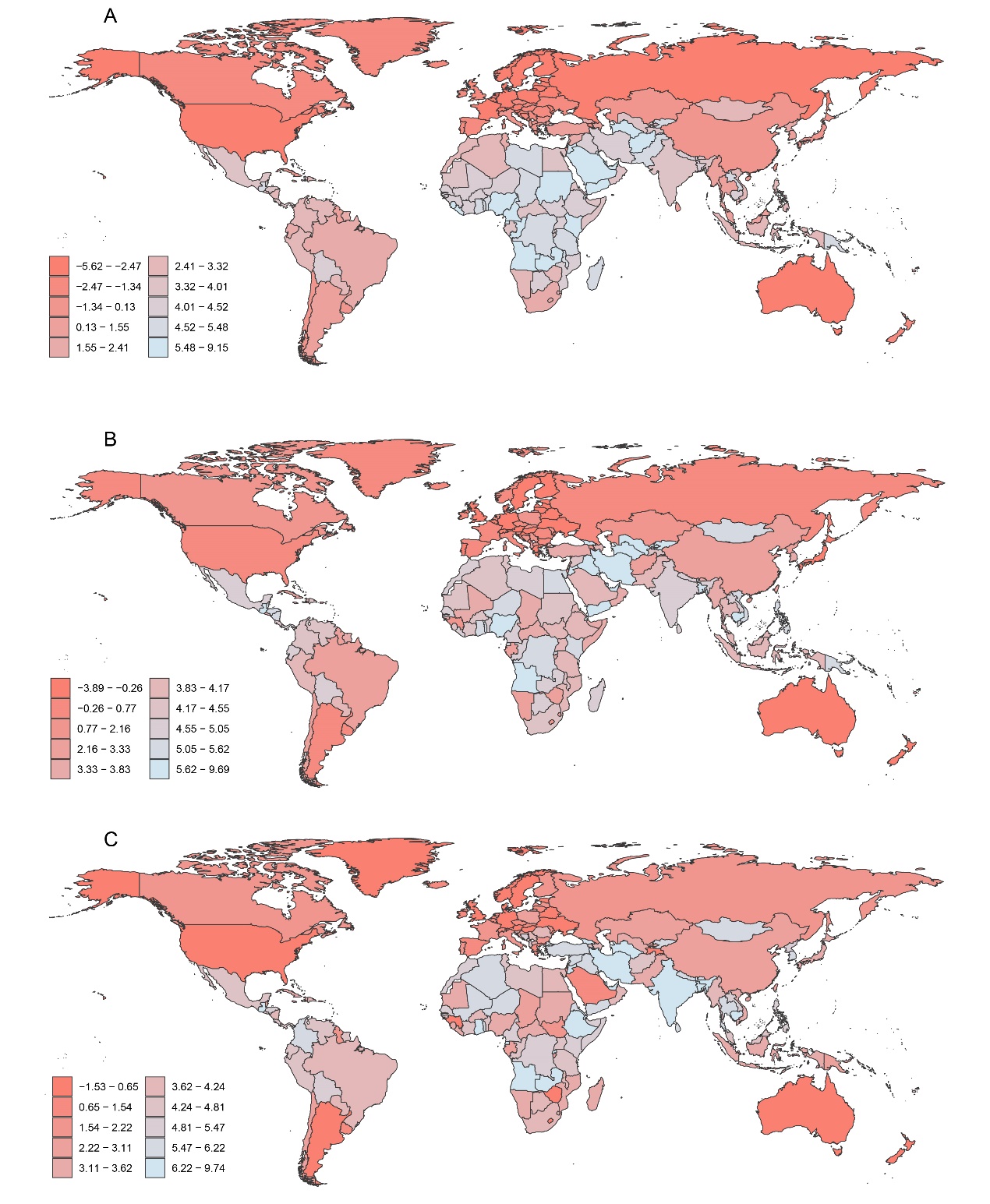


Figure S6 Global distribution of EAPC of ASDR for ovarian cancer among women of reproductive age, pre-elderly adults, and elderly adults in 2021.

A. The EAPC of ASDR for ovarian cancer in the reproductive age group. B. The EAPC of ASDR for ovarian cancer in pre-elderly adults group. C. The EAPC of ASDR for ovarian cancer in elderly adults group. EAPC: estimated annual percentage change; ASIR: age-standardized death rate; HSC: hormone-sensitive cancers.


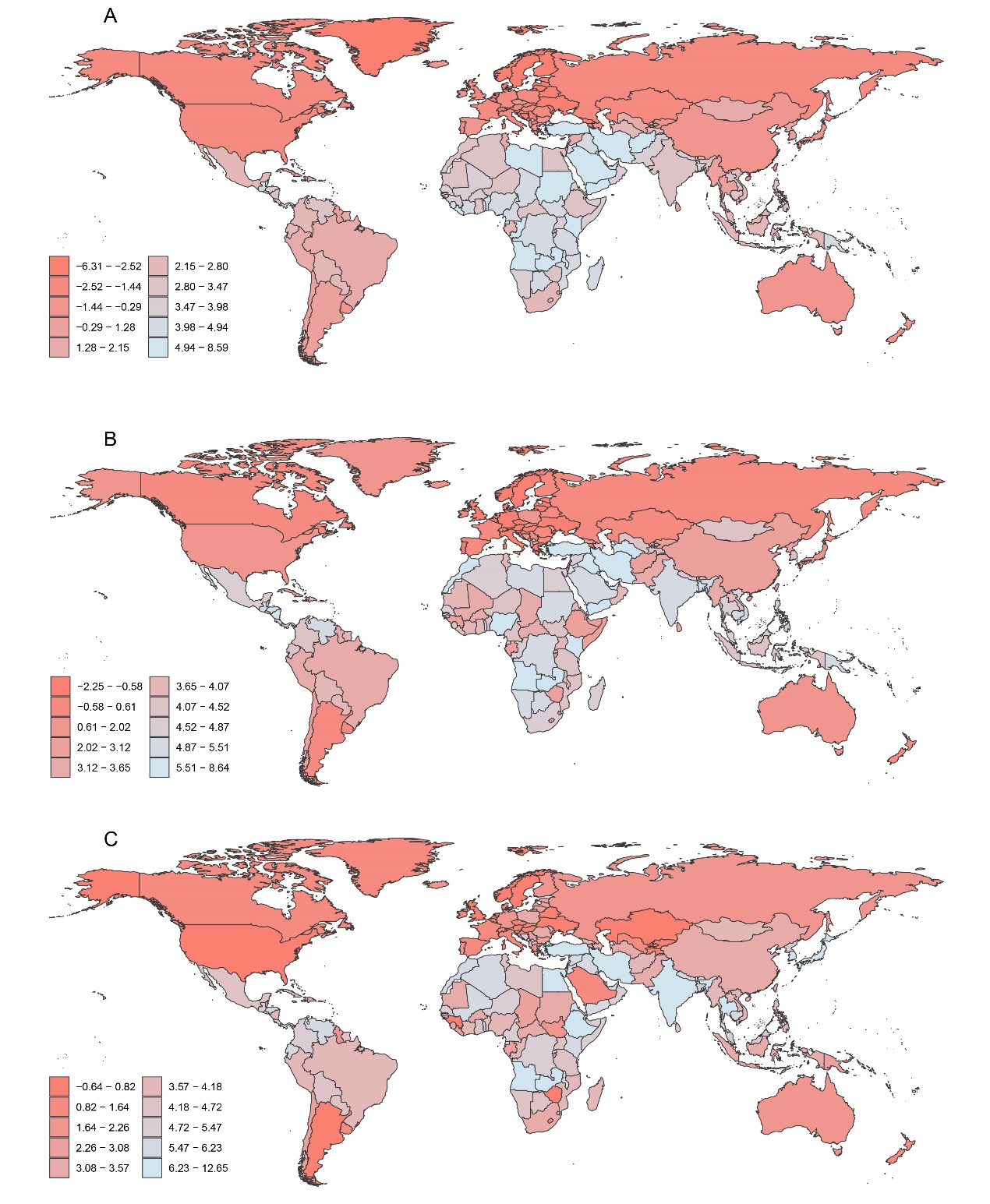


Figure S7 Global distribution of EAPC of ASDiR for breast cancer among women of reproductive age, pre-elderly adults, and elderly adults in 2021.

A. The EAPC of ASDiR for breast cancer in the reproductive age group. B. The EAPC of ASDiR for breast cancer in pre-elderly adults group. C. The EAPC of ASDiR for breast cancer in elderly adults group. EAPC: estimated annual percentage change; ASIR: age-standardized death rate; HSC: hormone-sensitive cancers.


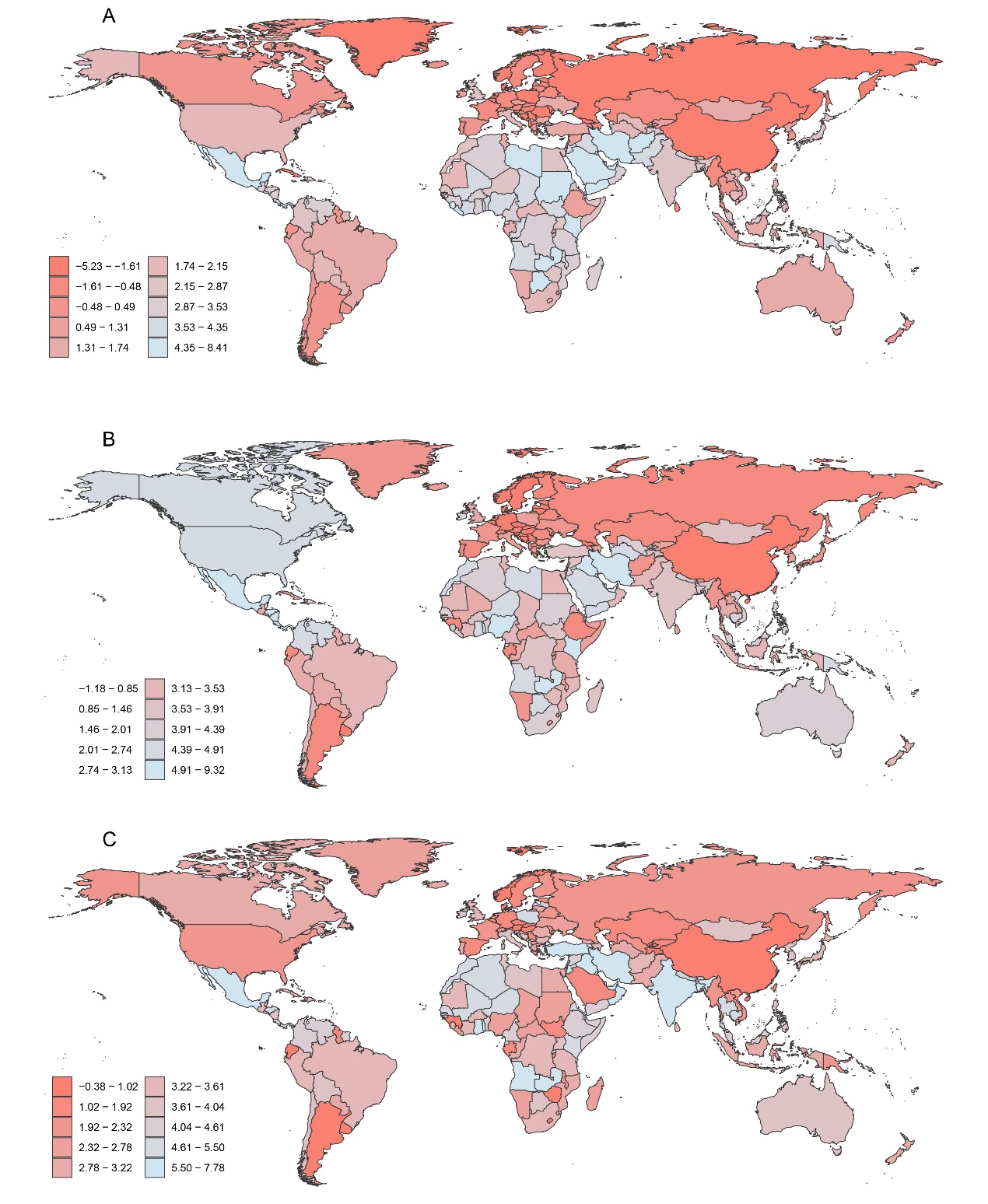


Figure S8 Global distribution of EAPC of ASDiR for uterine cancer among women of reproductive age, pre-elderly adults, and elderly adults in 2021.

A. The EAPC of ASDiR for uterine cancer in the reproductive age group. B. The EAPC of ASDiR for uterine cancer in pre-elderly adults group. C. The EAPC of ASDiR for uterine cancer in elderly adults group. EAPC: estimated annual percentage change; ASIR: age-standardized death rate; HSC: hormone-sensitive cancers.


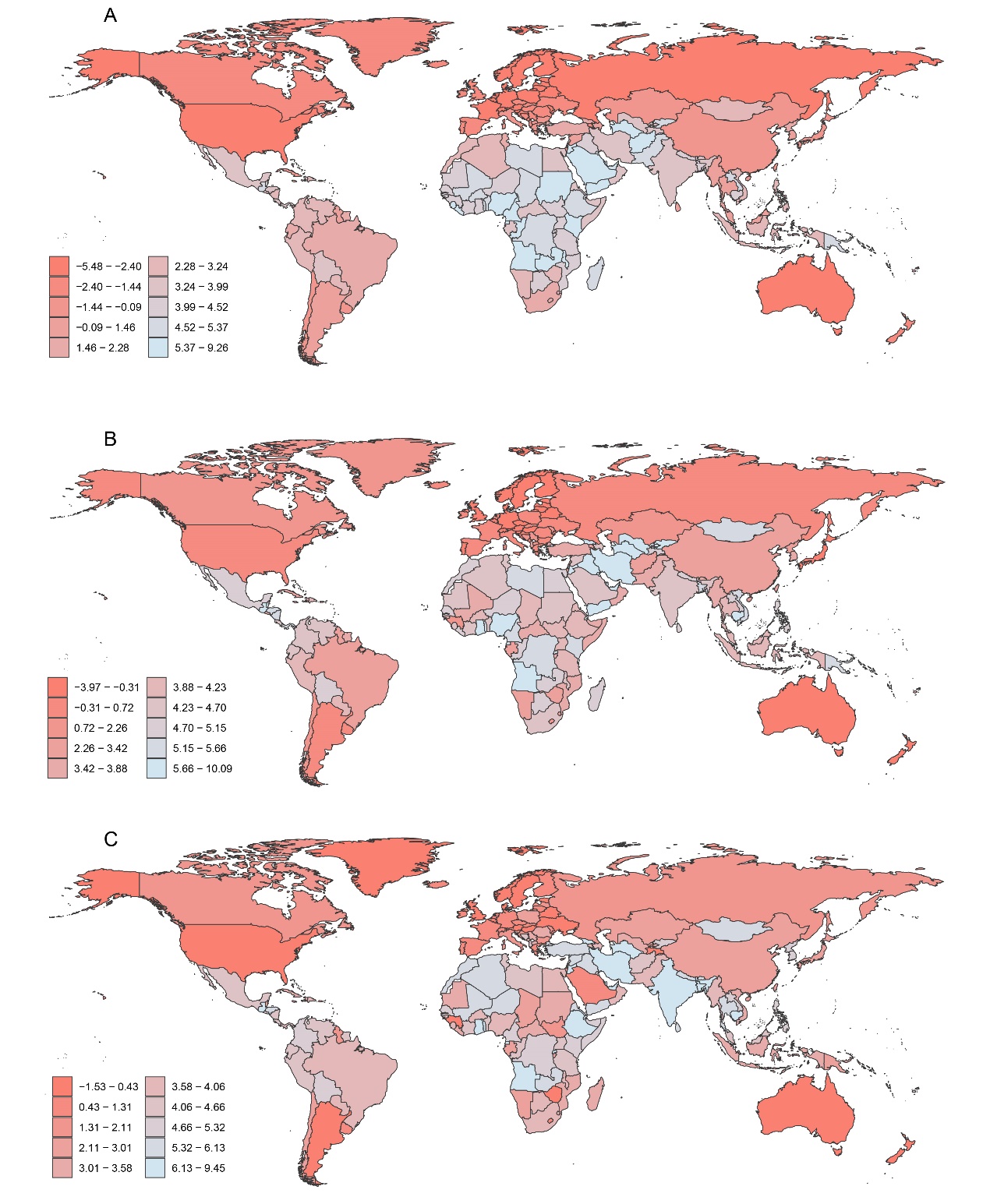


Figure S9 Global distribution of EAPC of ASDiR for ovarian cancer among women of reproductive age, pre-elderly adults, and elderly adults in 2021.

A. The EAPC of ASDiR for ovarian cancer in the reproductive age group. B. The EAPC of ASDiR for ovarian cancer in pre-elderly adults group. C. The EAPC of ASDiR for ovarian cancer in elderly adults group. EAPC: estimated annual percentage change; ASIR: age-standardized death rate; HSC: hormone-sensitive cancers.


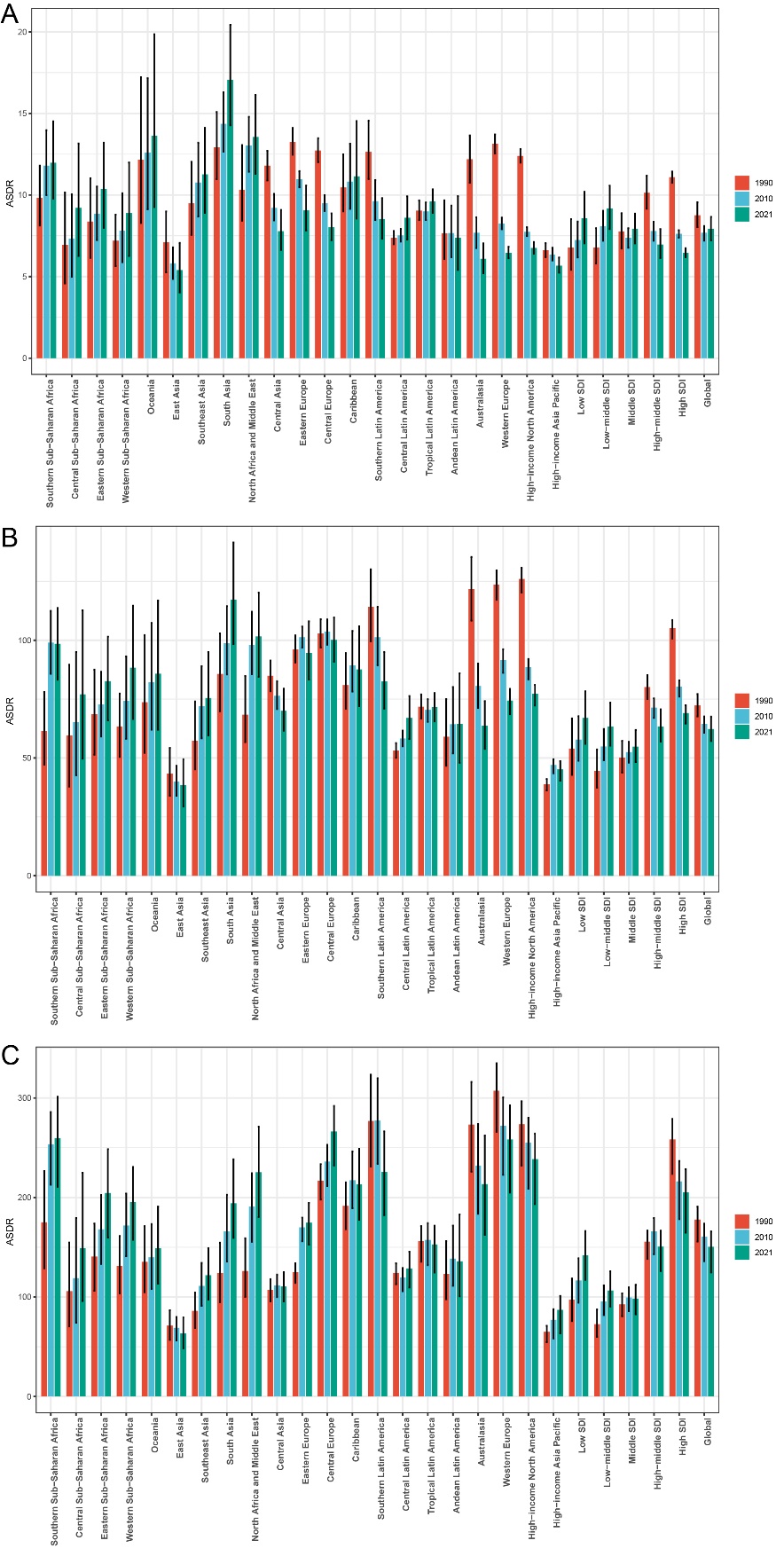


Figure S10 Regional ASDR barplot for HSC among women of reproductive age, pre-elderly adults, and elderly adults in 1990, 2010, and 2021.

A. The ASDR for HSC in the reproductive age group. B. The ASDR for HSC in pre-elderly adults group. C. The ASDR for HSC in elderly adults group. ASDR: age-standardized death rate; HSC: hormone-sensitive cancers.


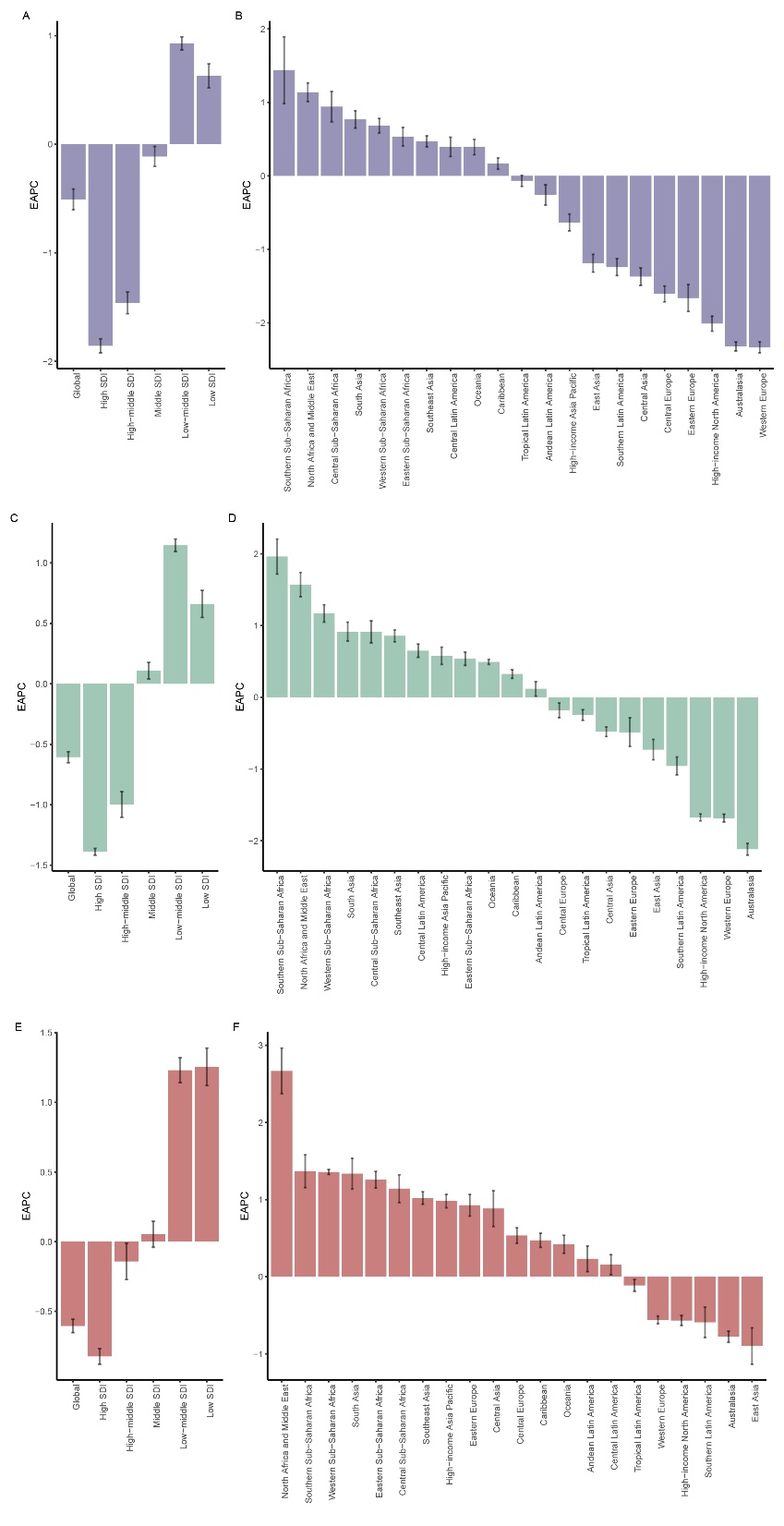


Figure S11 The EAPC of ASDR for HSC among women of reproductive age, pre-elderly adults, and elderly adults across 5 SDI quintiles and 21 GBD regions in 2021.

A. The EAPC of ASDR for HSC in the reproductive age group. B. The EAPC of ASDR for HSC in pre-elderly adults group. C. The EAPC of ASDR for HSC in elderly adults group. EAPC: estimated annual percentage change; ASDR: age-standardized death rate; HSC: hormone-sensitive cancers; SDI: socio-demographic index; GBD: global burden of disease.


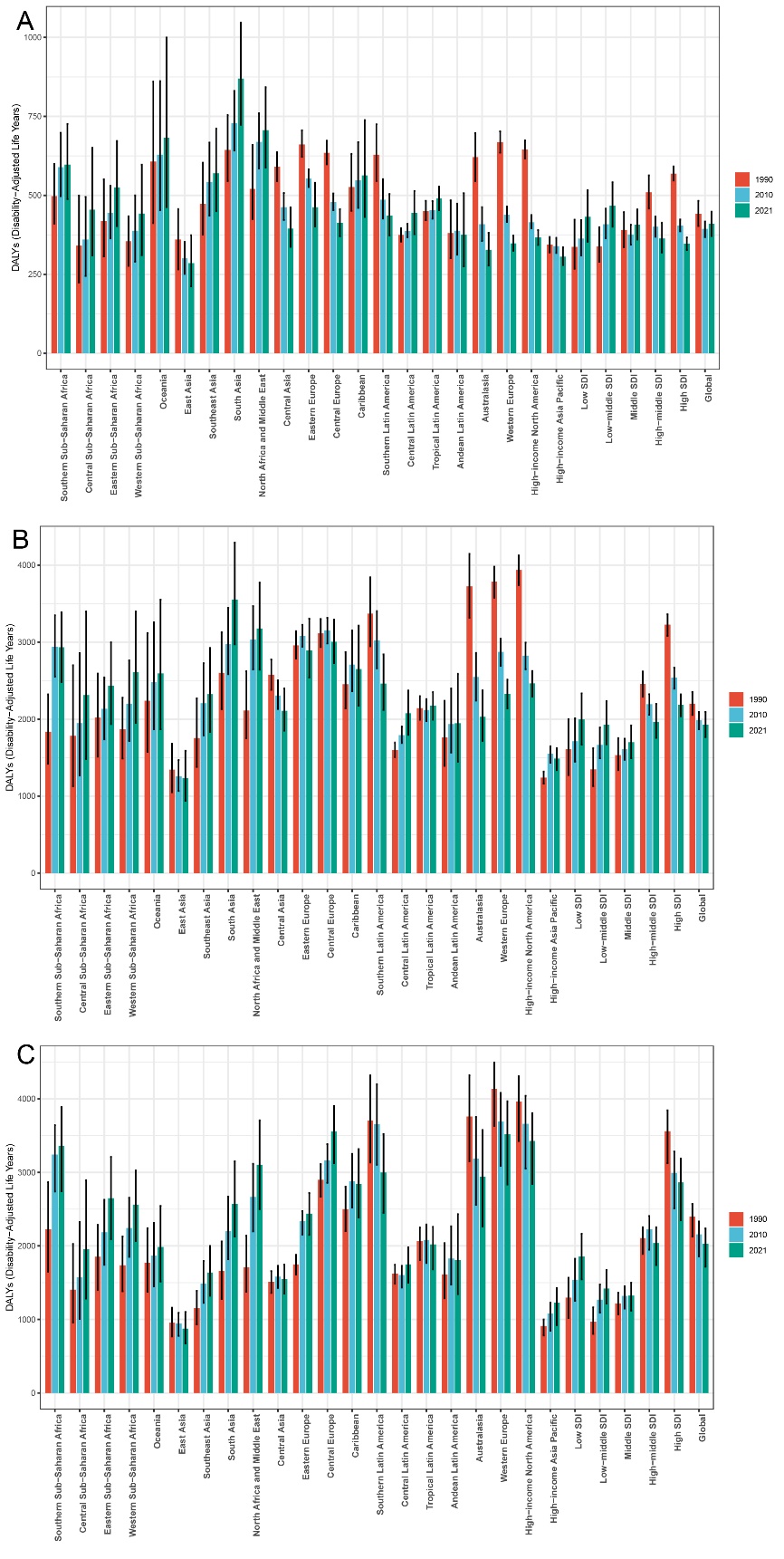


Figure S12 Regional ASDiR barplot for HSC among women of reproductive age, pre-elderly adults, and elderly adults in 1990, 2010, and 2021.

A. The ASDiR for HSC in the reproductive age group. B. The ASDiR for HSC in pre-elderly adults group. C. The ASDiR for HSC in elderly adults group. ASDiR: age-standardized disability-adjusted life years (DALYs) rate; HSC: hormone-sensitive cancers.


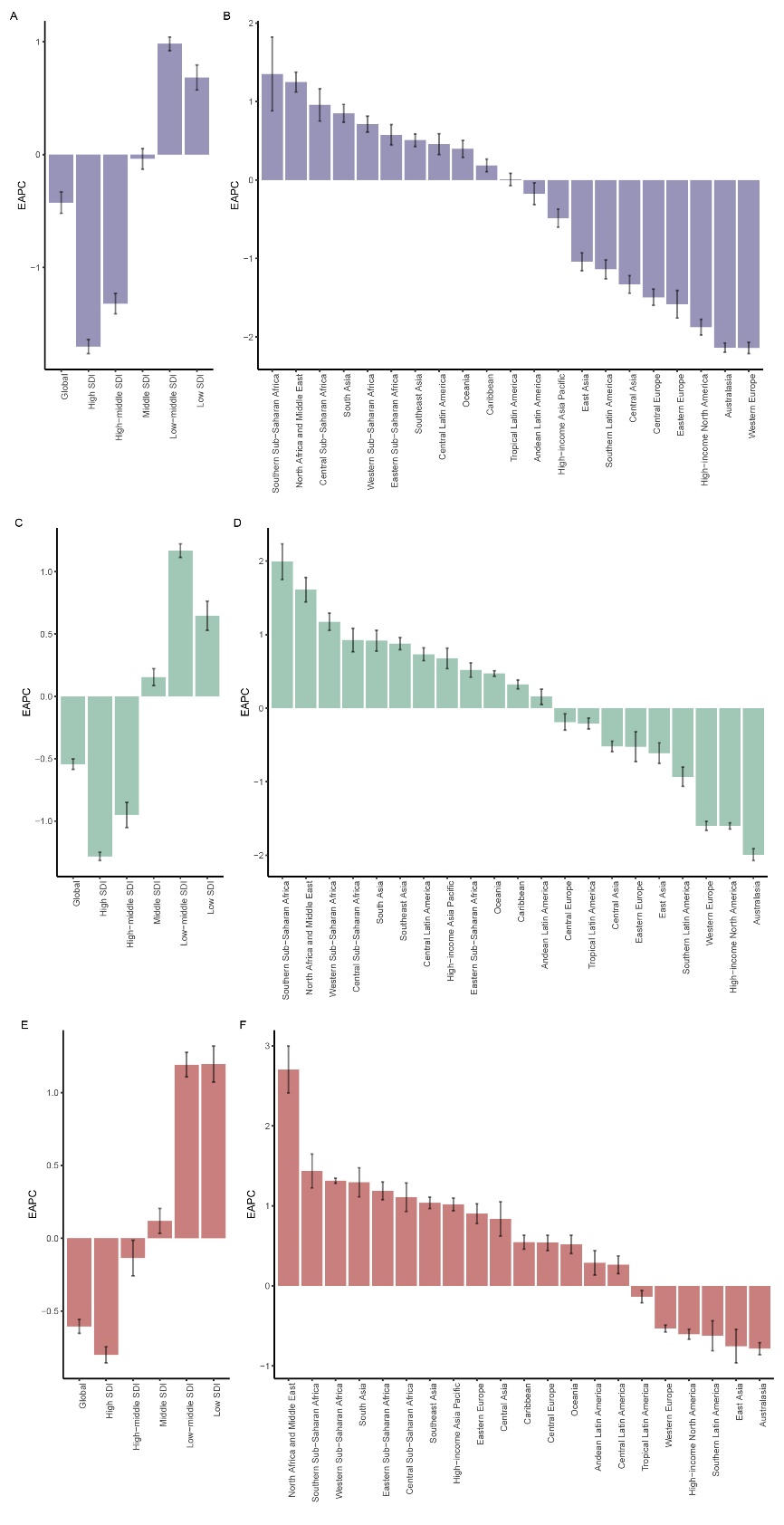


Figure S13 The EAPC of ASDiR for HSC among women of reproductive age, pre-elderly adults, and elderly adults across 5 SDI quintiles and 21 GBD regions in 2021.

A. The EAPC of ASDiR for HSC in the reproductive age group. B. The EAPC of ASDiR for HSC in pre-elderly adults group. C. The EAPC of ASDiR for HSC in elderly adults group. EAPC: estimated annual percentage change; ASDiR: age-standardized disability-adjusted life years (DALYs) rate; SDI: socio-demographic index; GBD: global burden of disease.


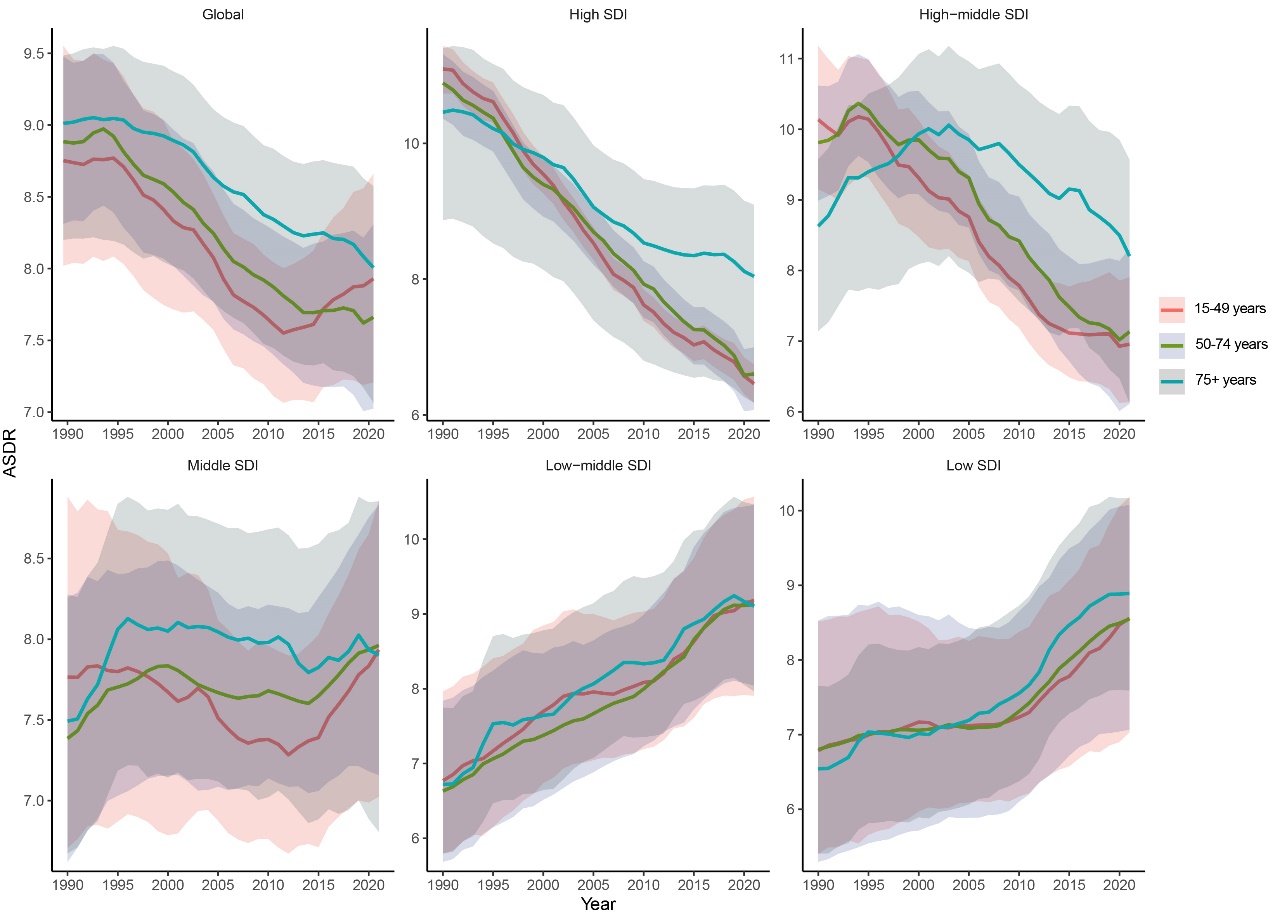


Figure S14 Time trend in ASDR of HSC among women of reproductive age, pre-elderly adults, and elderly adults across 5 SDI quintiles from 1990 to 2021.

ASDR: age-standardized death rate; HSC: hormone-sensitive cancers; SDI: socio-demographic index.


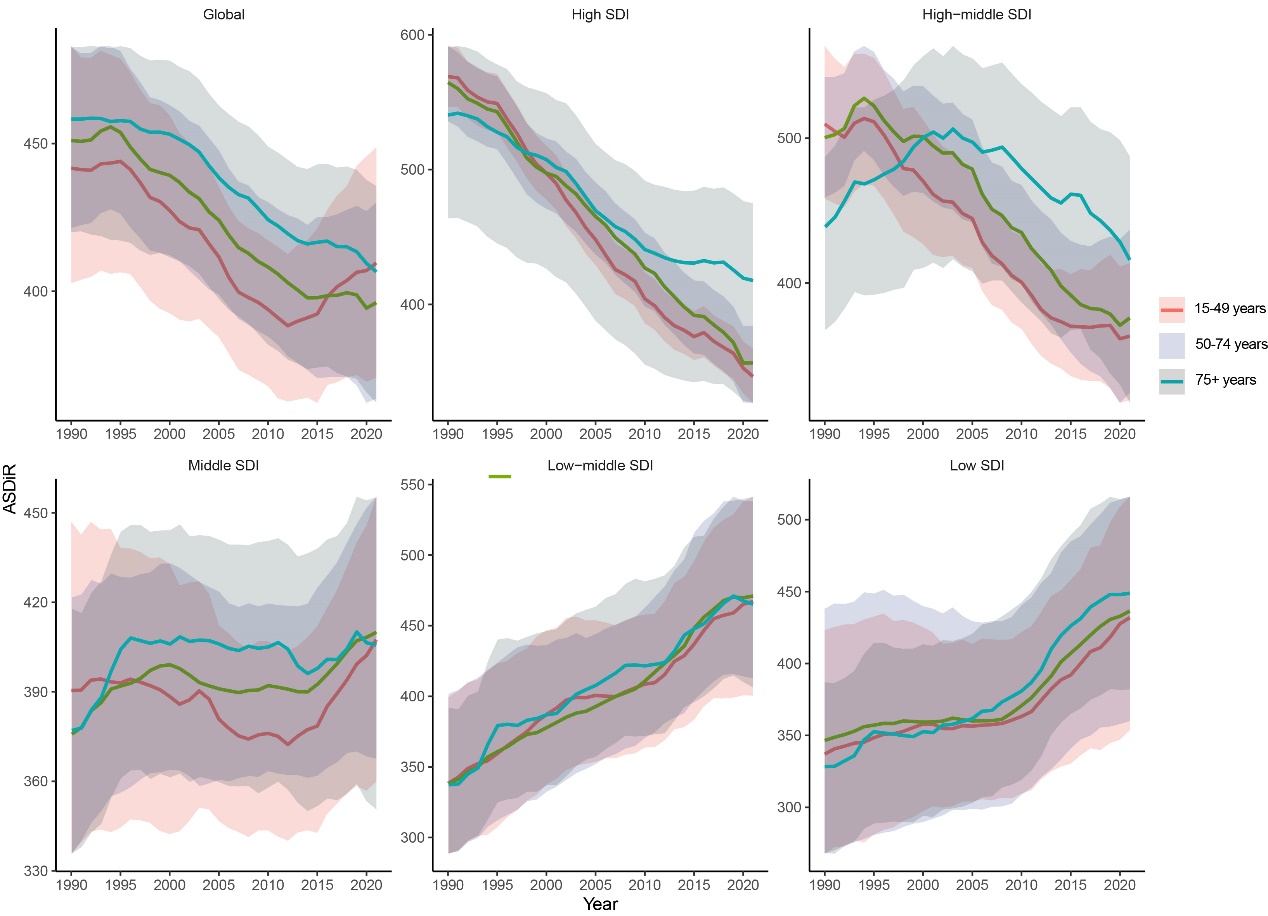


Figure S15 Time trend in ASDiR of HSC among women of reproductive age, pre-elderly adults, and elderly adults across 5 SDI quintiles from 1990 to 2021.

ASDiR: ASDiR: age-standardized disability-adjusted life years (DALYs) rate; HSC: hormone-sensitive cancers; SDI: socio-demographic index.


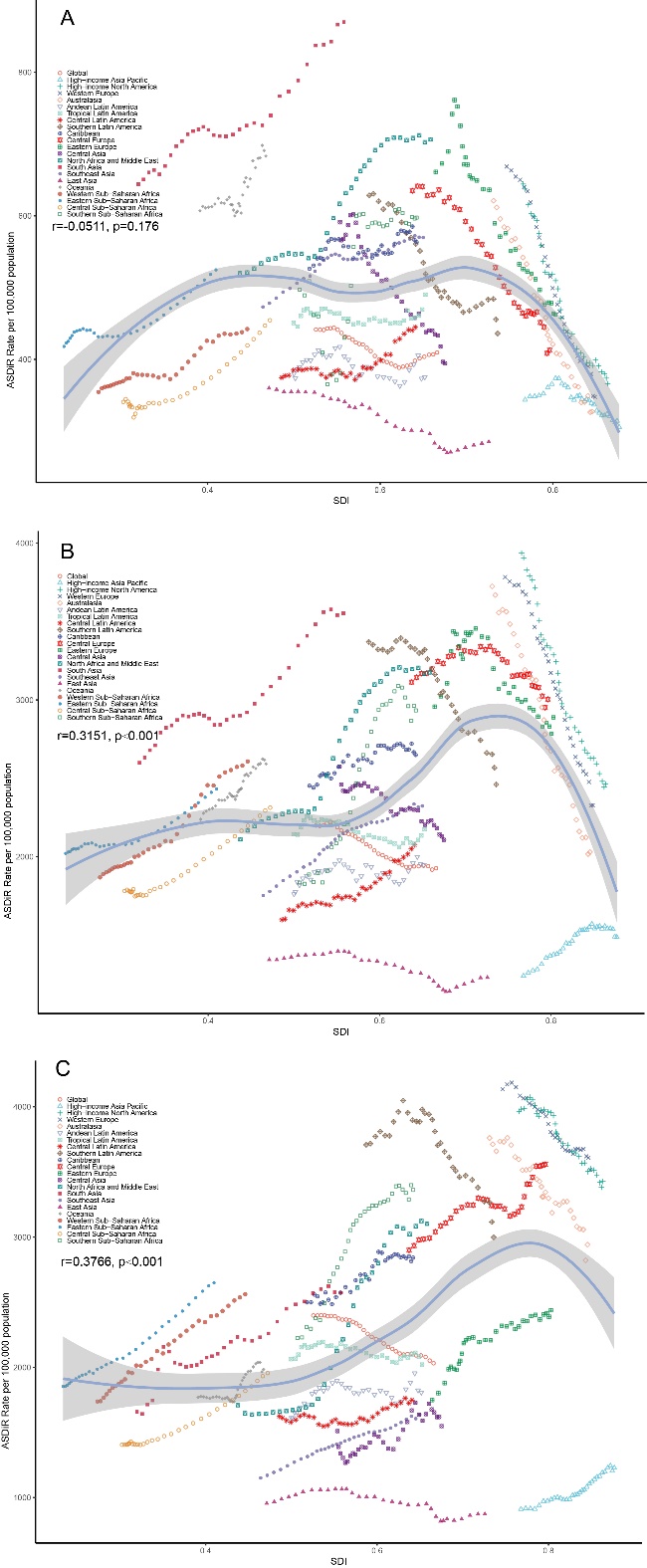


Figure S16 Spearman's correlation between ASDiR for HSC and SDI among women of reproductive age, pre-elderly adults, and elderly adults across 21 GBD regions, 1990 to 2021.

Each point represents the ASDiR corresponding to the SDI of the region in a given year, and the points are sequentially ordered from 1990 to 2021 from left to right. A: The correlation of ASDiR with SDI among women of reproductive age. B: The correlation of ASDiR with SDI among women of pre-elderly adults. C: The correlation of ASDiR with SDI among women of elderly adults. ASDiR: age-standardized disability-adjusted life years (DALYs) rate; HSC: hormone-sensitive cancers; SDI: socio-demographic index.


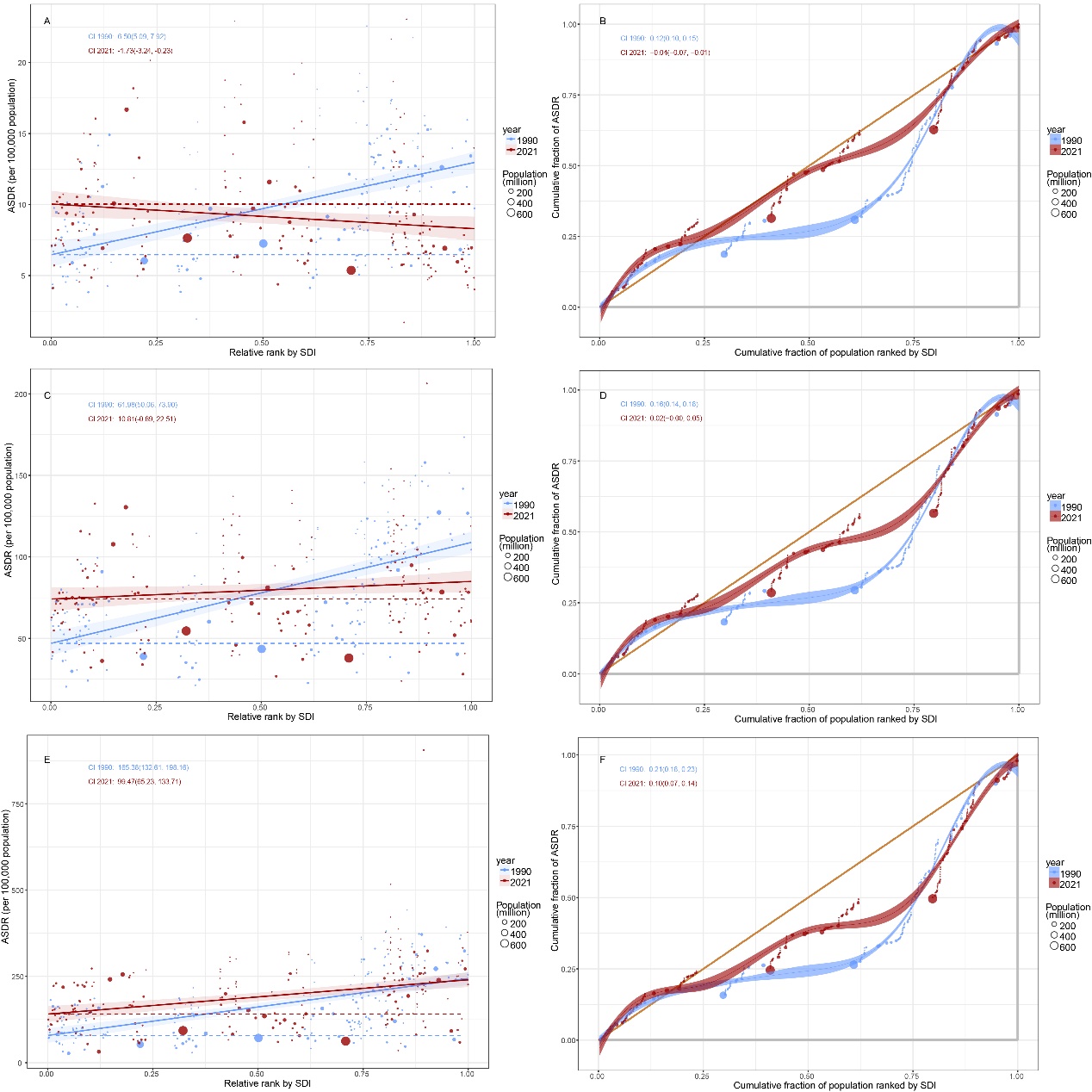


Figure S17 Inequality analysis of ASDR for HSC across all age groups in different countries/territories relative to their positions on the SDI scale, 1990 and 2021.

A, C, and E present the slope index of inequality for reproductive age, pre-elderly adults and elderly adults, respectively. B, D, and F represent the concentration index for reproductive age, pre-elderly adults and elderly adults, respectively. ASDR: age-standardized death rate; HSC: hormone-sensitive cancers; SDI: socio-demographic index.


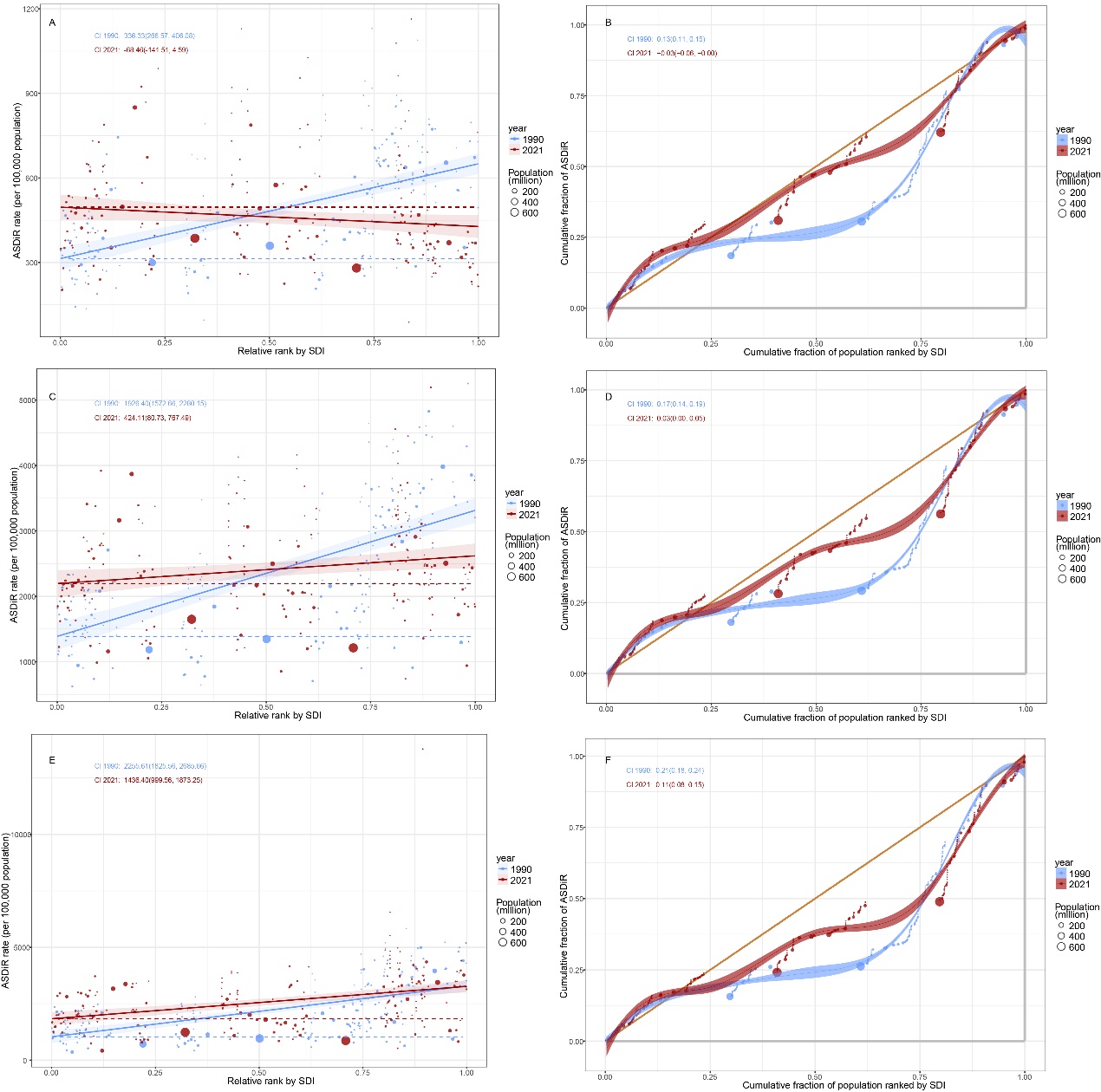


Figure S18 Inequality analysis of ASDiR for HSC across all age groups in different countries/territories relative to their positions on the SDI scale, 1990 and 2021.

A, C, and E present the slope index of inequality for reproductive age, pre-elderly adults and elderly adults, respectively. B, D, and F represent the concentration index for reproductive age, pre-elderly adults and elderly adults, respectively. ASDiR: age-standardized disability-adjusted life years (DALYs) rate; HSC: hormone-sensitive cancers; SDI: socio-demographic index.


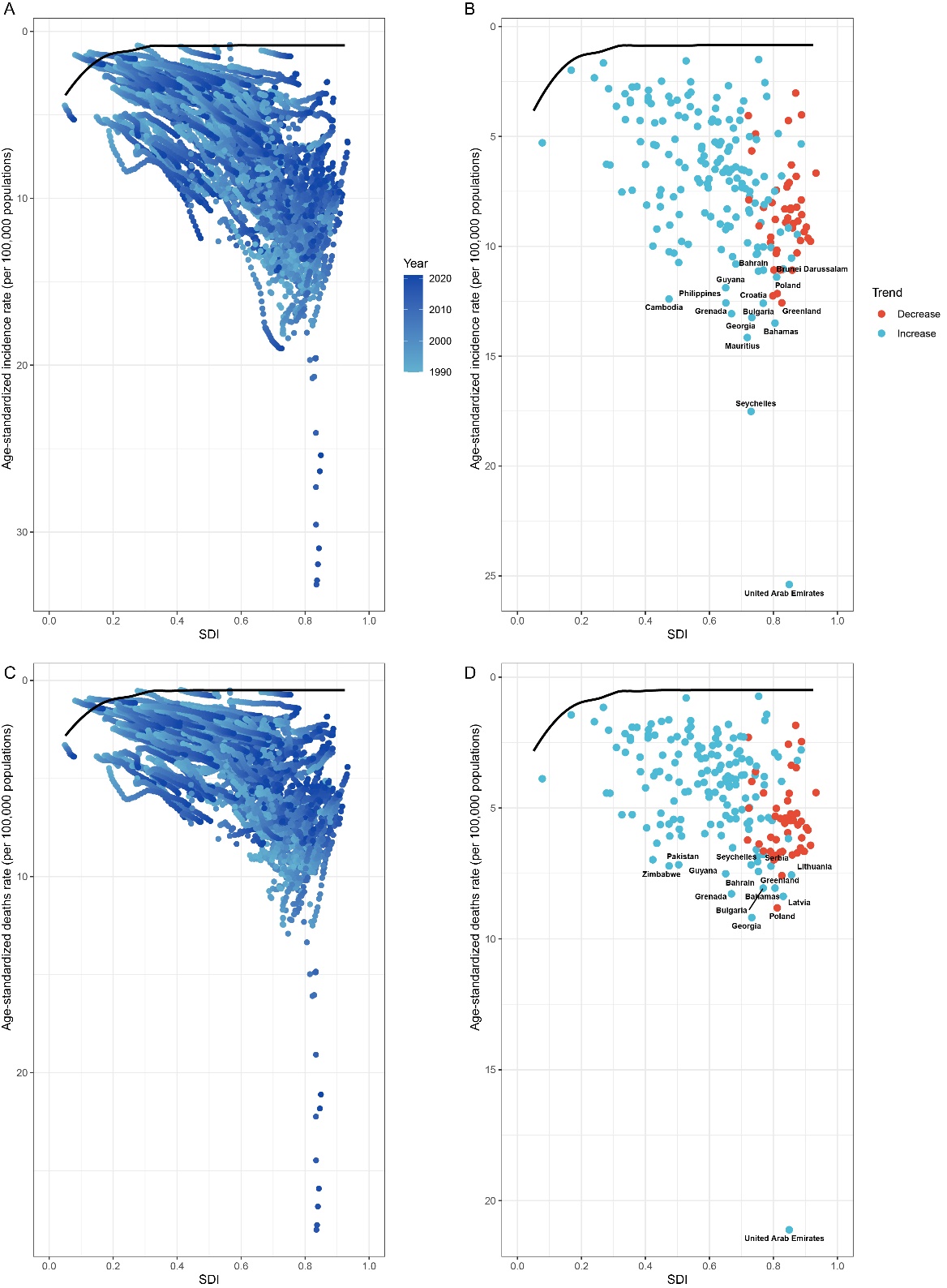


Figure S19 Frontier analysis based on SDI and ASIR/ASDR for ovarian cancer from 1990 to 2021.

A, C: represents the annual change in ASIR and ASDR for 204 countries/territories from 1990 to 2021, respectively, and the frontier line was generated in black solid. B, D: each dot represents the difference between a specific country/territory and the frontier line in 2021. The decrease in the differences between a country/territory and the frontier line from 1990 to 2021 is marked in blue, while the increase is shown in red. SDI: socio-demographic index; ASIR: age-standardized incidence rate; ASDR: age-standardized death rate.


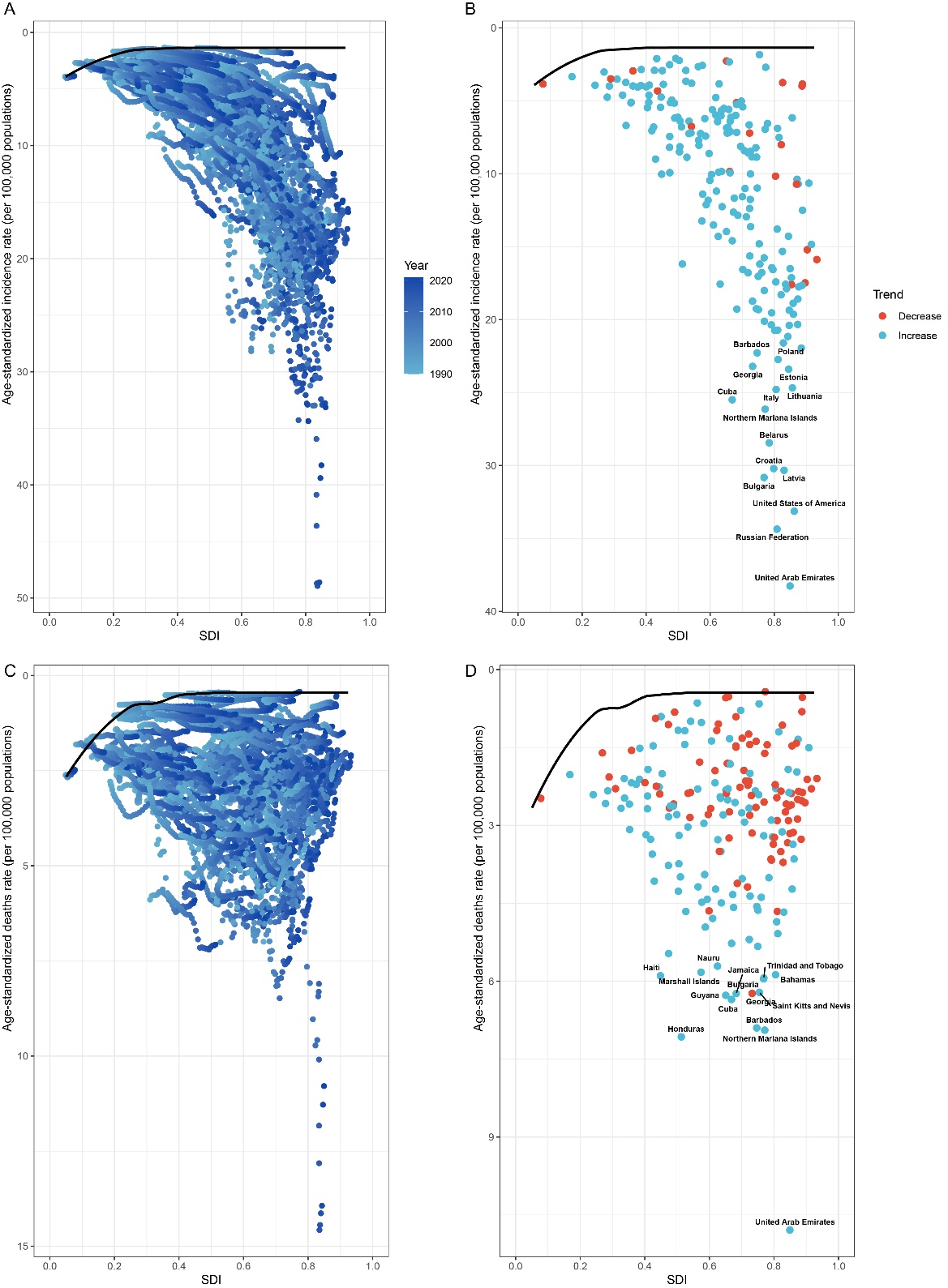


Figure S20 Frontier analysis based on SDI and ASIR/ASDR for uterine cancer from 1990 to 2021.

A, C: represents the annual change in ASIR and ASDR for 204 countries/territories from 1990 to 2021, respectively, and the frontier line was generated in black solid. B, D: each dot represents the difference between a specific country/territory and the frontier line in 2021. The decrease in the differences between a country/territory and the frontier line from 1990 to 2021 is marked in blue, while the increase is shown in red. SDI: socio-demographic index; ASIR: age-standardized incidence rate; ASDR: age-standardized death rate.


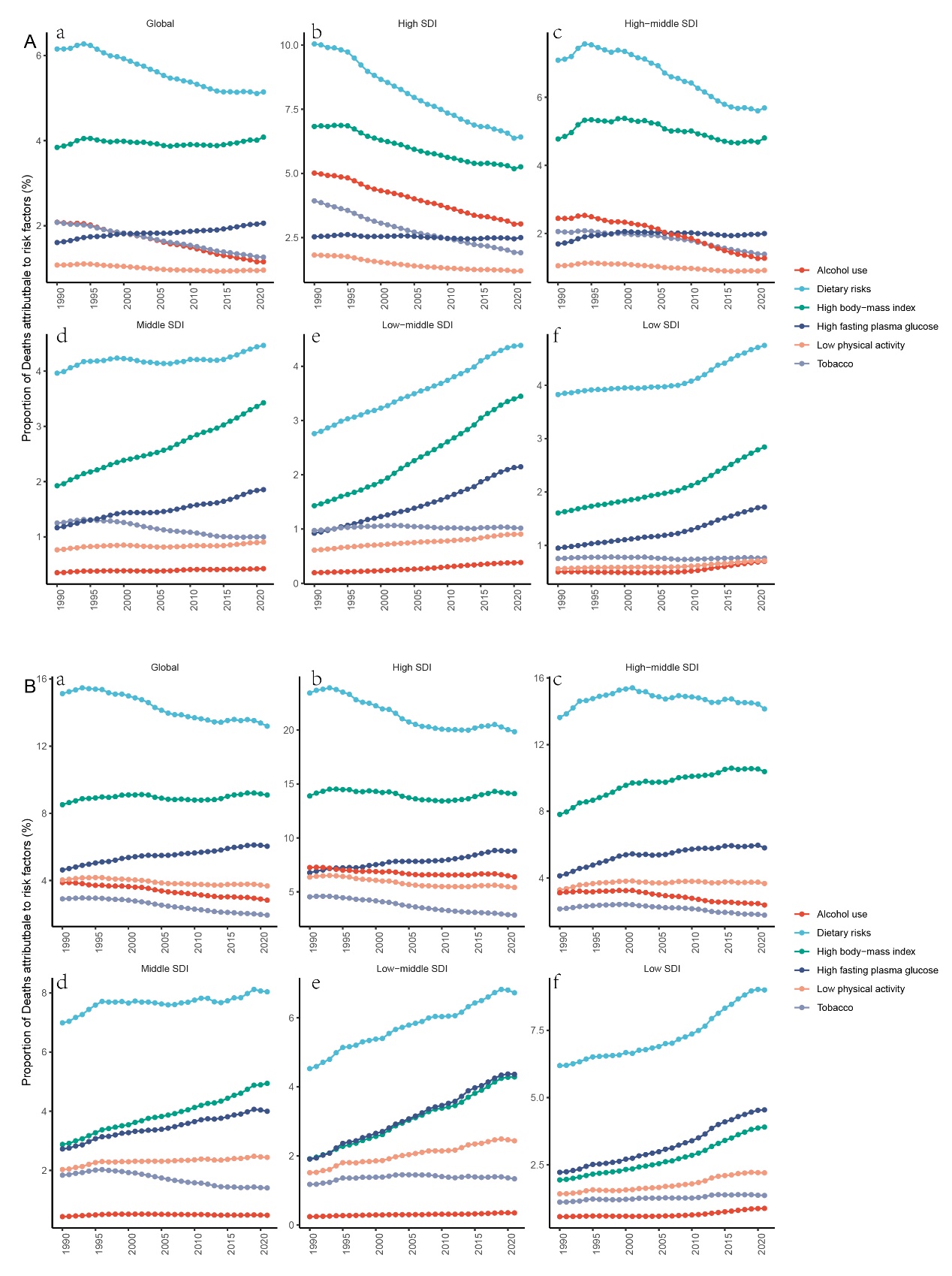


Figure S21 Trend of risk factors attributable to breast cancer ASDR among women of pre-elderly adults and elderly adults across the 5 SDI quintiles from 1990 to 2021.

ASDR: age-standardized death rate; SDI: socio-demographic index.


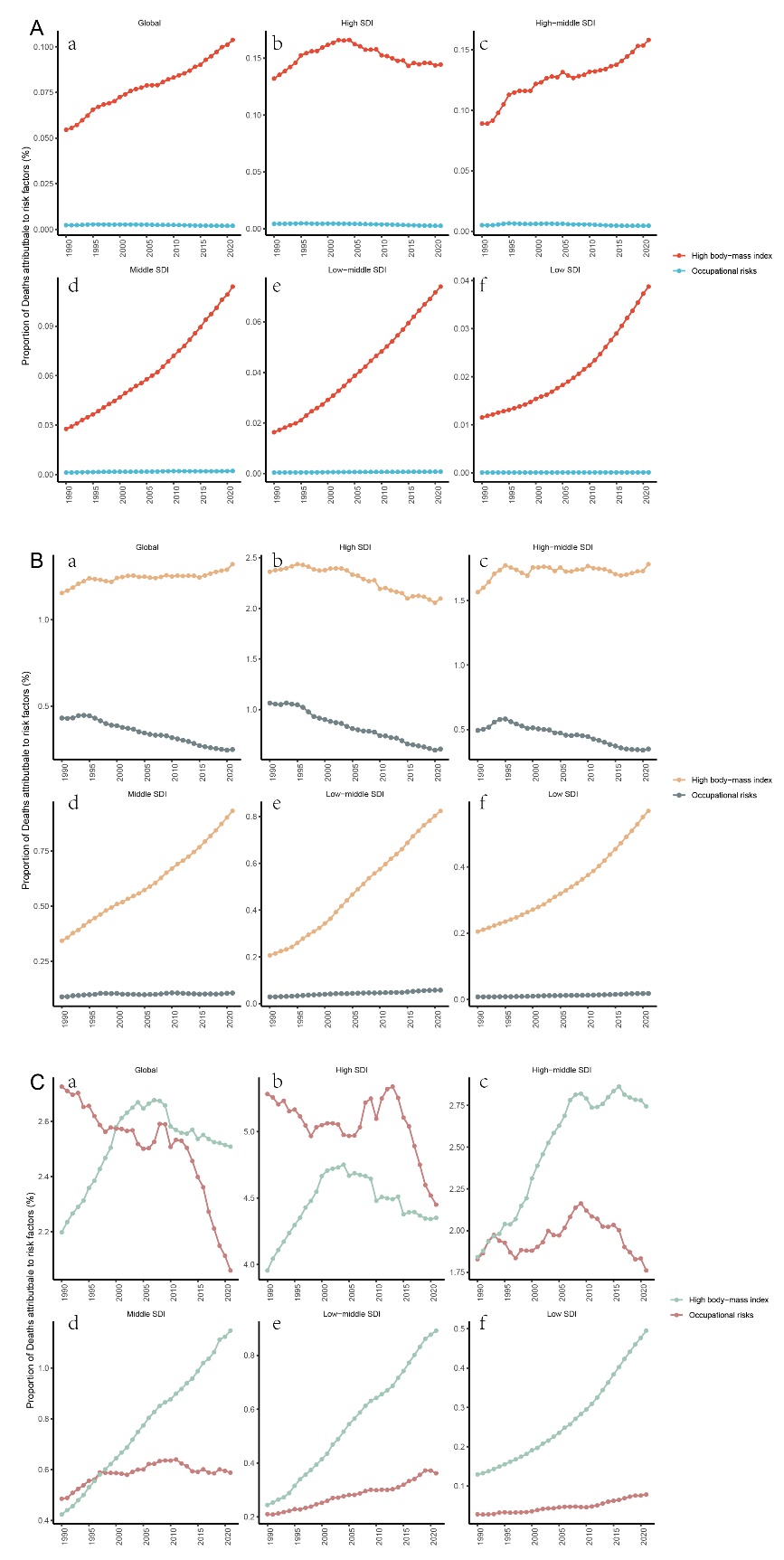


Figure S22 Trend of risk factors attributable to ovarian cancer ASDR among women of reproductive age, pre-elderly adults, and elderly adults age across the 5 SDI quintiles from 1990 to 2021.

ASDR: age-standardized death rate; SDI: socio-demographic index.


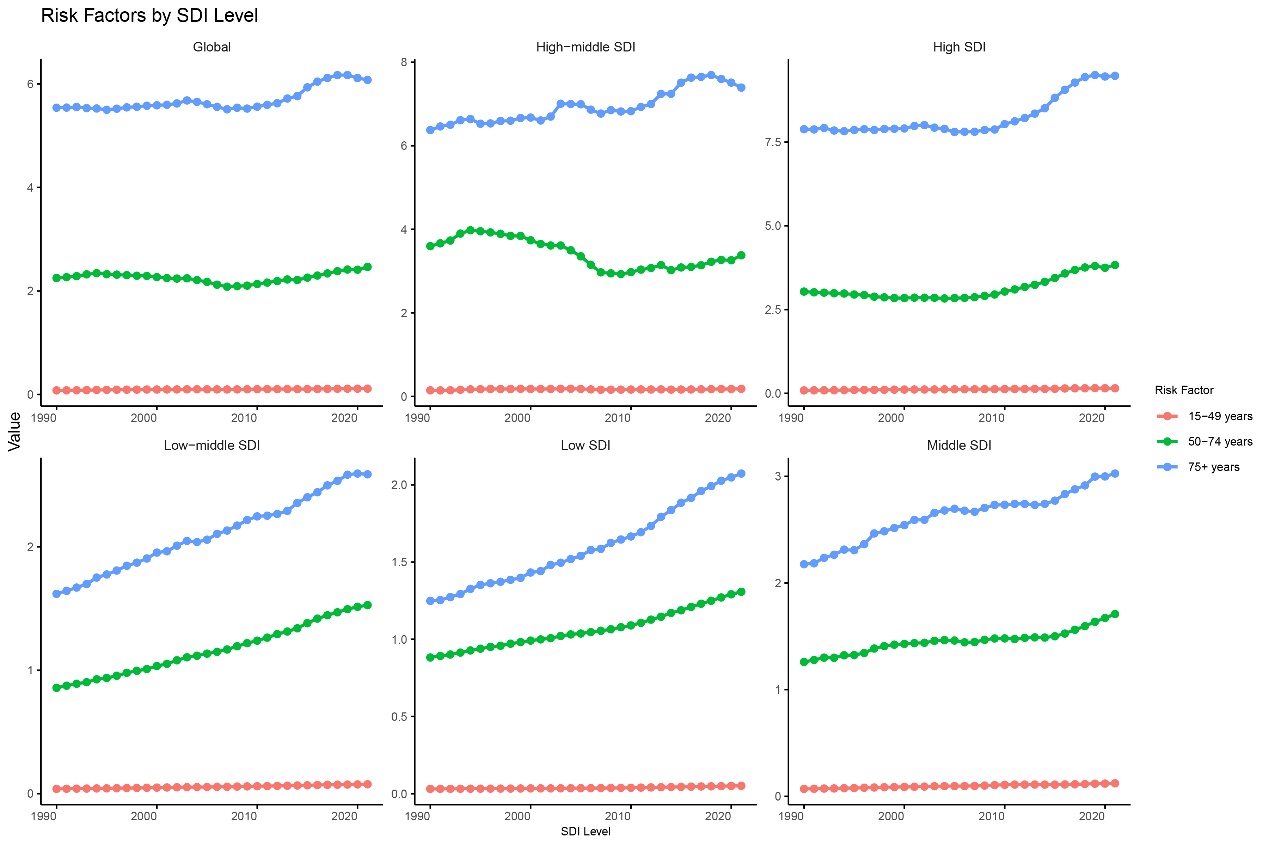


Figure S23 Trend of risk factors attributable to uterine cancer ASDR among women of reproductive age, pre-elderly adults, and elderly adults across the 5 SDI quintiles from 1990 to 2021.

ASDR: age-standardized death rate; SDI: socio-demographic index.
